# Key Clinical Interest Outcomes of Pharmaceutical Administration for Veterans With Post-Traumatic Stress Disorder Based on Pooled Evidences of 36 Randomised Controlled Trials With 2,331 Adults

**DOI:** 10.3389/fphar.2020.602447

**Published:** 2020-12-17

**Authors:** Yi-Fan Zhao, Zhen-Dong Huang, Hui-Yun Gu, Guang-Ling Guo, Rui-Xia Yuan, Chao Zhang

**Affiliations:** ^1^ Center for Evidence-Based Medicine and Clinical Research, Taihe Hospital, Hubei University of Medicine, Shiyan, China; ^2^ Southern Medical University, Guangzhou, China; ^3^ Department of Spine and Orthopedic Oncology, Zhongnan Hospital of Wuhan University, Wuhan, China; ^4^ Center of Women’s Health Sciences, Taihe Hospital, Hubei University of Medicine, Shiyan, China; ^5^ Clinical Big Data Center, The First Affiliated Hospital of Zhengzhou University, Zhengzhou, China

**Keywords:** post-traumatic stress disorder, Veteran, risperidone, sertraline, PTSD symptoms scale

## Abstract

**Background:** The effects of drug treatment on veterans, who have a high risk of post-traumatic stress disorder (PTSD), are not clear, and the guidelines are different from the recommendations of the recent meta-analysis. Our goal was to find the efficacy and frequencies of complications of drugs that can treat PTSD in veterans.

**Method: **We searched Ovid MEDLINE, Ovid Embase, The Cochrane Library and Web of Science until January 1, 2020. The outcomes were designed as the change of PTSD total scale, subsymptom score, response rate, frequencies of complications outcomes, and acceptability.

**Results:** We included a total of 36 randomised controlled trials with a total of 2,331 adults. In terms of overall effect, drug treatment is more effective than placebo in change in total PTSD symptoms scale (SMD = −0.24, 95% CI [−0.42, −0.06]) and response (RR = 1.66, 95% CI [1.01, 2.72]). However, in terms of frequencies of complications, drugs generally had a higher withdrawal rate (RR = 1.02, 95% CI [0.86, 1.20]) and a higher frequencies of complications (RR = 1.72, 95% CI [1.20, 2.47]) than placebo. Risperidone showed a good curative effect in change in total PTSD symptoms scale (SMD = −0.22, 95% CI [−0.43, 0.00]) and acceptability (RR = 1.31, 95% CI [0.82, 2.59]). The drugs acting on 5−HT receptors, our results showed that symptoms of hyper−arousal (SMD = −0.54, 95% CI [−0.86, −0.21]), symptoms of re−experiencing (SMD = −0.62, 95% CI [−0.86, −0.39]) and symptoms of avoidance (SMD = −0.53, 95% CI [− 0.77,−0.3]), The drugs acting on dopamine receptors, our results showed that symptoms of re−experiencing (SMD = −0.35, 95% CI [−0.55, −0.16]) and the drugs acting on α2 receptor has a significant effect on reducing total PTSD symptoms scale (SMD = −0.34, 95% CI [−0.62, −0.06]).

**Conclusion:** Drug therapy can effectively treat PTSD, but its frequencies of complications should be considered. Different from the guidelines for adult PTSD, this study supports atypical antipsychotics, selective serotonin reuptake inhibitors and receptors that act on 5-HT and dopamine for the treatment of PTSD in veterans. Based on evidence among these drugs, the risperidone is the most effective for veterans, otherwise, sertraline is used as an alternative.

## Introduction

Post-traumatic stress disorder (PTSD) is a type of the mental disorder that is difficult to treat at present. This is a common mental health consequence of exposure to extreme, life-threatening stress/serious injury, it is characterized by the presence of the following 4 clusters of symptoms according to the Diagnostic and Statistical Manual of Mental Disorders, Fourth Edition (DSM-IV) criteria: re-experiencing symptoms, persistent avoidance, negative alterations in cognition and mood and hyperarousal (APA, 2013). It may lead to severe depression or other mental illness, and may even lead to physiological complications, such as diabetes and cardiovascular disease (Koenen et al., 2017; Scherrer et al., 2020). The sick population’s years of life lost (YLLs) and disability-adjusted life-years (DALYs) have a great influence (Murray et al., 2013). However, studies had shown that the cost of treating PTSD may be as high as tens of thousands of dollars (Ivanova et al., 2011). More than 500,000 American veterans spend billions of dollars seeking treatment (IOM, 2014), and the treatment cycle is long. Therefore, as a kind of disease that does great harm to people and is expensive to treat, an effective treatment method for PTSD has been a topic of concern for many scholars.

The treatment of PTSD mainly includes drug therapy, psychotherapy and adjuvant therapy. Related studies (Charney et al., 1993) had showed that PTSD has long been associated with monoamine neurotransmitters. Therefore, drug treatment is possible. American Psychological Association (APA) and National Institute for Health and Care Excellence (NICE) guidelines (Wynn, 2015; Association, 2017) and some studies have indicated that psychotherapy is effective (Gerger et al., 2014; Belsher et al., 2019), but the effect of drug treatment is not satisfactory. The Food and Drug Administration (FDA) of the United States currently only approves sertraline and paroxetine for the treatment of PTSD, while APA guidelines recommend fluoxetine, paroxetine, sertraline and venlafaxine (Association, 2017). According to the NICE guidelines, only paroxetine, mirtazapine, amitriptyline and phenylethyl were significantly superior to placebo (Health, 2005). However, a study on drugs in the treatment of PTSD suggest that drugs, such as selective serotonin reuptake inhibitors (SSRIs) and serotonin-norepinephrine reuptake inhibitors (SNRIs), can be used as first-line treatment (Koirala et al., 2017). Research is developing new drugs, such as oxytocin (Flanagan et al., 2019). Another study also suggested that the current results of drug treatment are uncertain because the results of clinical studies are contradictory (Petrakis & Simpson, 2017). At present, drug treatment is very controversial, and in terms of the prevalence of PTSD, the prevalence of PTSD among civilians is 5.7%, while that of veterans is estimated at 30.9% (Dohrenwend et al., 2006). Related studies (Richardson et al., 2010) had showed that the prevalence of PTSD in veterans can be as high as 17% in the first year of deployment. PTSD symptoms caused by combat-related events in veterans are typically more severe than those experienced by non-veterans, and treatment efficacy was less than that in non-veterans (Goodson et al., 2011). At present, there are no relevant studies focusing on veterans, we carried out meta-analysis for veterans, who are susceptible to PTSD. In this meta-analysis, we not only evaluated the effectiveness and frequencies of complications of various drug interventions for veterans, but also analyzed the gender differences and co-disease differences among veterans, providing the latest evidence for people in urgent need of effective treatment.

## Method

### Search Strategy

All studies had obtained by searching the Ovid Medline, EMBase, The Cochrane Library and ISI Web of Science for articles that were published until January 1, 2020. Two reviewers (YFZ and ZDH) independently assessed the abstracts and potentially eligible articles identified during literature selection. Discrepancies were resolved in discussions. If necessary, a final reviewer (CZ) was involved when faced with a disagreement. Detailed search strategies are shown in Supplementary Method 1. The guideline from the Preferred Reporting Items for Systematic Review and Meta-Analyses (PRISMA) statement (McInnes et al., 2018) was employed for this meta-analysis.

### Study Selection

Two reviewers (YFZ and ZDH) independently assessed abstracts and potentially eligible articles identified during literature selection. Discrepancies were resolved by discussion. If necessary, a final reviewer (CZ) was involved when faced with disagreement.

The following inclusion criteria were used: 1) Patients: the patients were diagnosed as PTSD by DSM-IV, DSM-III and DSM-IV-TR (APA, 2013); all patients had combat-related PTSD, and more than 95% of the total population in the study were veterans. Patients with comorbid psychosis were not excluded (including alcohol use disorder, material dependence, etc.); 2) Intervention: the main research intervention includes drugs, regardless of the mode of administration; 3) Control: single active control drug or placebo; 4) Outcome: contains at least one interesting outcome, including change in total PTSD symptoms scale, response, frequencies of complications, acceptability, avoidance, hyper-arousal and re-experiencing. Change in total PTSD symptoms scale, response, frequencies of complications and acceptability were chosen as the primary outcomes. Subsymptoms, including avoidance, hyper-arousal and re-experiencing, were chosen as the secondary outcomes; 5) Study design: randomised controlled trial (RCT).

The following exclusion criteria were used: 1) There is no data in the original research or the data cannot be converted. 2) Trials involving recurrent population studies or interventions to prevent PTSD in advance. 3) Duplicate publication. 4) Intervention differs from control by two or more drugs (excluding premedication that had no effect on outcome). 5) Psychotherapy is included in the intervention.

### Data Extraction and Quality Assessment

Information and data were extracted by two independent authors (YFZ and ZDH), proofreading and conflict resolution was handled by a final investigator (CZ). With regard to the selection of scales for symptom relief in PTSD, the priority scale evaluated by clinicians is a self-rated scale. The Clinically Administered PTSD Scale (CAPS) score is the gold standard scale evaluated by clinicians (Weathers et al., 2018). For self-rated PTSD, the Davidson Trauma Scale was used as the gold standard (Zuromski et al., 2019). If the gold standard scale is not used in the study, other scales are used. We chose the difference before and after change in value for comparison; if not, we used the final measured value (Higgins & Green, 2011). If a study does not report the data at the end of the study, then we will choose the most recent data after the end of the study. All the outcome data were analyzed by intention-to-treat (ITT) analysis as far as possible (Gupta, 2011), to ensure the accuracy of the data.

On the quality evaluation of included studies, two reviewers (YFZ and ZDH) independently assessed the quality of the included studies based on the risk of bias of Cochrane’s handbook (Higgins & Green, 2011).

### Statistical Analysis

When analyzing the difference of the binary data, risk ratios (RR) with 95% confidence interval (CI) is used as the effect amount. When analyzing differences in continuous data, we used standardized mean difference (SMD) with 95% CI (Friedrich et al., 2011). If the SD value was not reported directly in the study, we will make appropriate statistical conversion according to the Cochrane’s manual to obtain the value (Higgins & Green, 2011).

With regard to the heterogeneity of the study, we used I^2^ to measure it (Higgins et al., 2003). The random effect model was used when I^2^ > 40% (Lipsey, 2001), and the fixed effect model was used when I^2^ ≤ 40%. The guidelines (Wynn, 2015; Association, 2017) and various studies (Koirala et al., 2017; Flanagan et al., 2019) have different views on drugs to treat PTSD. This may be because there are many differences in PTSD patients, including gender differences. To assessed the potential differences from confounding factors, we divided participants into the following subgroups, including the previous medication, gender (Broidy et al., 2015), acting receptor, service battlefield (Kang et al., 2003; Hoge et al., 2006) and whether they had comorbid psychosis other than PTSD. To evaluate the effectiveness of specific drugs, we analyzed the use of drugs recommended (risperidone and sertraline) in the previous guidelines. Funnel plots were used to determine publication bias, and at least 10 studies were involved in each outcome to ensure accuracy (Duval & Tweedie, 2000). The R 3.5.1 software was employed for all data statistics.

## Result

### Search Results

**FIGURE 1:**
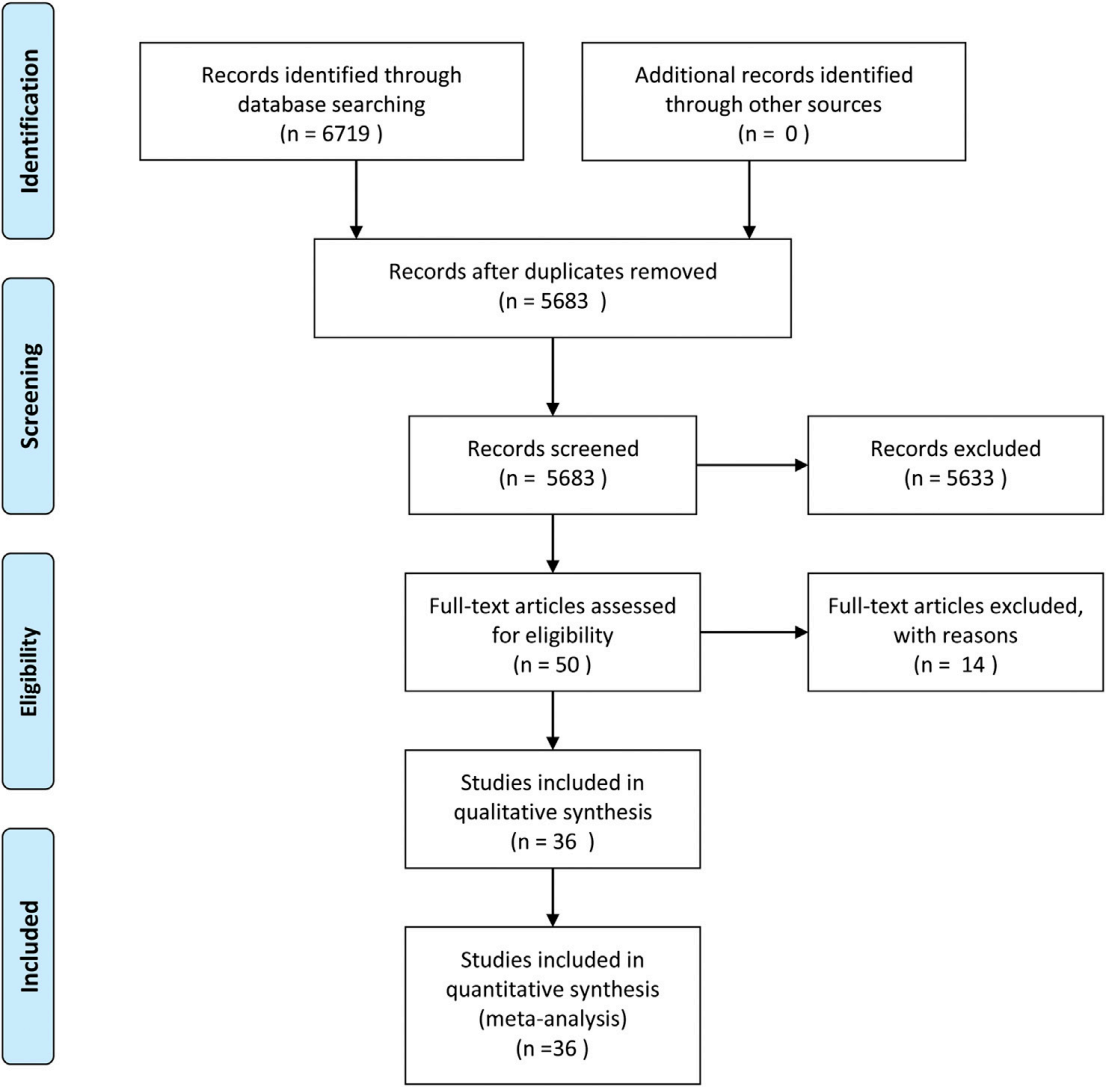
Search strategy.

Ovid Medline, Ovid EMBASE, The Cochrane Library and Web of Science were systematically searched until January 1, 2020. The search resulted in 6,719 articles. After initial evaluation, 1,036 studies were removed for being duplicates, 5,633 for being irrelevant (as determined by reading the title and abstracts) and 36 studies for reasons determined by reading the full text.ccc Figure 1 shows the work flow for the selection of studies.

### Study Characteristics

Characteristics of 2,331 adult patients from published double-blind, parallel 36 RCTs (Frank et al., 1988; Davidson et al., 1990; Kosten et al., 1991; Hertzberg et al., 2000; Stein et al., 2002; Zohar et al., 2002; Hamner et al., 2003; Monnelly et al., 2003; Akuchekian & Amanat, 2004; Chung et al., 2004; Davis et al., 2004; Bartzokis et al., 2005; Neylan et al., 2006; Friedman et al., 2007; Lindley et al., 2007; Raskind et al., 2007; Davis et al., 2008a; Davis et al., 2008b; Hamner et al., 2009; Krystal et al., 2011; Panahi et al., 2011; Germain et al., 2012; Litz et al., 2012; Baniasadi et al., 2014; Batki et al., 2014; Manteghi et al., 2014; Rothbaum et al., 2014; Naylor et al., 2015; Back et al., 2016; Petrakis et al., 2016; Rodgman et al., 2016; Villarreal et al., 2016; Ramaswamy et al., 2017; Rezaei Ardani et al., 2017; Surís et al., 2017; Raskind et al., 2018) were described in the study. Among them, three studies (Frank et al., 1988; Kosten et al., 1991; Rothbaum et al., 2014) reported active-comparator experiments. Our research involves a variety of drug types, including atypical antipsychotics (AASs) (Stein et al., 2002; Monnelly et al., 2003; Bartzokis et al., 2005; Hamner et al., 2009; Krystal et al., 2011; Naylor et al., 2015; Villarreal et al., 2016), corticosteroids (Surís et al., 2017), alpha blockers (Raskind et al., 2007; Germain et al., 2012; Petrakis et al., 2016; Rodgman et al., 2016; Raskind et al., 2018), anticonvulsants (Hamner et al., 2003; Akuchekian & Amanat, 2004; Lindley et al., 2007; Davis et al., 2008a; Batki et al., 2014), central muscle relaxants (Manteghi et al., 2014), D-cycloserine (Litz et al., 2012; Rothbaum et al., 2014), N-acetylcysteine (Back et al., 2016), γ-aminobutyric acid (Baniasadi et al., 2014), reversible cholinesterase inhibitor (Rezaei Ardani et al., 2017), serotonin antagonist and reuptake inhibitors (SARIs) (Davis et al., 2004); SSRIs (Hertzberg et al., 2000; Zohar et al., 2002; Friedman et al., 2007; Panahi et al., 2011; Ramaswamy et al., 2017), tricyclic antidepressants (TCAs) (Frank et al., 1988; Davidson et al., 1990; Kosten et al., 1991), α2 receptor agonist (Chung et al., 2004; Neylan et al., 2006; Davis et al., 2008b), pregabalin (Baniasadi et al., 2014) and monoamine oxidase inhibitors (MAOIs) (Frank et al., 1988; Kosten et al., 1991). All patients were diagnosed as PTSD by different versions of the DSM scale. Only one study (Davis et al., 2004) included one civilian, and the other studies exclusively included veterans. In 15 studies, there was more than one intervention drug, or patients were likely to use other drugs. The population included in 15 studies was entirely comprised of males. Thirty studies used the clinician-assessed scale as the main outcome, and 26 of them used the CAPS scale. Summary estimates from the meta-analyses are presented in Table 1.

**TABLE 1:** Study Characteristics of including studies.

Study	Participants (I/C)	Age	Male (%)	Race (white)	During of treatment (week)	Baseline score	Population	Type of trauma	Diagnostic criteria	Intervention/control	Duration of illness (years)
Akuchekian and Amanat (2004)	34/33	39.80 (4.19)	67 (100%)	NR	12	CAPS: 50.70 (7.70)/48.90 (9.13)	Veterans	All combat-related	DSM-IV	Topiramate (50–500 mg/day vs. placebo	17.90 (2.20)
Back et al. (2016)	13/14	49.00 (8.20)	26 (96%)	8	8	CAPS: 58.80 (21.20)/68.60 (23.70); PCL: 45.70(14.60)/43.40(18.60)	Veterans	Military (combat5,non-combat 9),civilian-related events 13	DSM-IV	Fixed dose n-acetylcysteine 2,400 vs. placebo	NR
Baniasadi et al. (2014)	18/19	48.16 (3.55)	37 (100%)	NR	6	PCL: 56.83(7.66)/55.10(7.75)	Veterans	All combat-related	DSM-IV-TR	Pregabalin 75–300 vs. placebo	28.37 (1.81)
Bartzokis (2005)	33/32	51.60 (4.20)	65 (100%)	44	16	CAPS: 102.20 (11.90)/98.60 (15.80)	Veterans	All combat-related	DSM-IV	Risperidone 1–3 mg vs. placebo	NR
Batki et al. (2014)	14/16	49.98 (13.10)	28 (93%)	16	12	CAPS: 72.80(14.30)/83.10(17.30)	Veterans	22 combat-related	DSM-IV-TR	Topiramate (25–300) vs. placebo	NR
Chung et al. (2004)	51/49	59.10 (6.00)	100 (100%)	NA	6	CAPS: 103.20(22.40)/88.80(23.90)	Veterans	All combat-related	DSM-IV	15 mg mirtazapine vs. 50 mg sertraline	33.50 (10.60)
Davidson et al. (1990)	25/21	49.22 (11.94)	NR	NR	8	IES: 33.10 (8.50)/36.80 (5.30)	Veterans	All combat-related	DSM-III	Amitriptyline 158.3 + 91.7 (50–300) vs. placebo	NR
Davis et al. (2004)	26/15	53.80 (8.10)	40 (98%)	22	12	CAPS: 81.00 (20.00)/83.20 (17.00); PCL: 64.10(11.00)/61.50(10.00)	Veterans (40), Civilian (1)	Combat-related 40 (97.5%)	DSM-IV	Nefazodone (100–600) vs. placebo	29.86 (12.74)
Davis et al. (2008a)	44/41	55.20 (6.80)	83 (98%)	NR	8	CAPS: 75.20(19.10)/77.30(15.30)	Veterans	78 combat-related	DSM-IV	Divalproex 2,309 + 507 (1,000–3,000) vs. placebo	24.40 (10.90)
Davis et al. (2008b)	18/17	53.46 (7.46)	32 (91%)	25	8	CAPS: 82.06(16.81)/88.41(17.87); DTS: 92.33(22.50)/100.77(27.74)	Veterans	All combat-related	DSM-IV	Guanfacine (1–2 mg/d) vs. placebo	NR
Frank et al. (1988)	12/11	38 (11.70)	23 (100%)	NR	8	IES: 40.00(14.20)/41.00(12.90)	Veterans	All combat-related	DSM III-R	imipmamine300 mg/dversus phenelzine	NR
Frank et al. (1988)	11/11	38.00 (11.70)	22 (100%)	NR	8	IES: 41.00(12.90)/35.00(12.30)	Veterans	All combat-related	DSM III-R	phenelzine15–75 mg/d vs. placebo	NR
Frank et al. (1988)	12/11	38.00 (11.70)	23 (100%)	NR	8	IES: 40.00(14.20)/35.00(12.30)	Veterans	All combat-related	DSM III-R	imipmamine300 mg vs. placebo	NR
Friedman et al. (2007)	86/83	45.32 (10.31)	135 (80%)	120	12	CAPS: 72.10 (19.10)/73.80 (19.80); IES: 40.70(15.80)/43.40(15.60)	Veterans	120 combat-related	DSM-III-R	Sertraline (25–200 mg/day) vs. placebo	18.30 (12.13)
Germain et al. (2012)	18/15	40.00 (14.10)	33 (100%)	29	8	PCL: 43.90 (17.30)/35.00 (14.10)	Veterans	All combat-related	DSM-IV	Prazosin 1 mg vs. placebo	NR
Hamner et al. (2003)	19/18	52.21 (6.44)	NR	17	5	CAPS: 90.30 (23.00)/89.10 (12.20)	Veterans	All combat-related	DSM-IV	Risperidone 29.2 + 9.7 (13–45) vs. placebo	NR
Hamner et al. (2009)	16/13	52.38 (6.89)	28 (97%)	26	10	CAPS: 76.67 (23.80)/77.62 (21.91); IES: 44.33(19.49)/42.42(14.48)	Veterans	Combat (n = 28) and sexual Assault (n = 1)	DSM-IV	Divalproex 1,196 + 246 mg (500–1,500) vs. placebo	NR
Hertzberg et al. (2000)	6/6	46.00 (r:44–48)	12 (100%)	5	12	DTS: 106.00(27.00)/111.00(12.00)	Veterans	All combat-related	DSM-IV	Fluoxetine 48 (10–60) vs. placebo	NR
Kosten et al. (1991)	19/23	39.00 (1.98)	42 (100%)	39	8	IES: 30.60(15.20)/36.50(16.70)	Veterans	All combat-related	DSM III	Imipramine 225 (50–300) vs. phenelzine 68 mg (15–75)	NR
Kosten et al. (1991)	19/18	38.51 (2.04)	37 (100%)	31	8	IES: 30.60(15.20)/33.00(13.40)	Veterans	All combat-related	DSM III	Imipramine 225 (50–300) vs. placebo	NR
Kosten et al. (1991)	23/18	38.56 (2.04)	41 (100%)	34	8	IES: 36.50(16.70)/33.00(13.40)	Veterans	All combat-related	DSM III	Phenelzine 68 mg (15–75)vs. placebo	NR
Krystal et al. (2011)	133/134	54.40 (10.70)	258 (97%)	117	24	CAPS: 78.20 (15.00)/78.20 (14.70); PCL: 64.10(10.60)/63.60(11.70)	Veterans	209 combat-related events	DSM-IV	Risperidone (1–4) vs. placebo	NR
Lindley et al. (2007)	20/20	53.40 (0.76)	40 (100%)	25	7	CAPS: 62.10 (12.90)/61.00 (22.20)	Veterans	All combat-related	NR	Topiramate (50–200) vs. placebo	NR
Litz et al. (2012)	13/13	32.19 (9.31)	26 (100%)	20	6	CAPS: 69.85(23.24)/73.38(16.35); PCL: 37.85(8.76)/39.00(8.77)	Veterans	All combat-related	DSM-IV	50 mgD-cycloserine vs. placebo	NR
Manteghi et al. (2014)	20/20	46.61 (9.31)	40 (100%)	NA	8	CAPS: 61.00(16.06)/61.80(14.38)	Veterans	All combat-related	NA	Baclofen 10 mg/d-40 mg/d in 3 divided daily vs. placebo	NR
Monnelly et al. (2003)	7/8	51.35 (6.30)	15 (100%)	NR	6	PCL: 73.00(NR)/72.00(NR)	Veterans	All combat-related	DSM-IV	Risperidone 0.57 + 0.13 (0.5–2) mg vs. placebo	NR
Naylor et al. (2015)	7/7	33.82 (4.81)	9 (64%)	7	10	CAPS: 90.60 (10.32)/82.29(19.1); PCL: 60.57(4.51)/60.50(5.46)	Veterans	All combat-related	DSM-IV	Aripiprazole 10 (5–20)vs. placebo	NR
Neylan et al. (2006)	29/34	NR	NR	NR	8	CAPS: 67.10(20.60)/69.40(20.80)	Veterans	All combat-related	DSM-IV	Guanfacine 2.4 + 0.7 (0.5–3) vs. placebo	NR
Panahi et al. (2011)	35/35	45.55 (5.30)	70 (100%)	NR	10	IES: 65.40(3.90)/65.10(5.00)	Veterans	Male iranian veterans with combat-related PTSD	DSM-IV-TR	Sertraline 140 + 33 (50–200)vs. placebo	24.10 (2.77)
Petrakis et al. (2016)	50/46	43.97 (13.02)	89 (93%)	78	13	CAPS: 71.86(20.32)/75.86(14.44)	Veterans	All combat-related	DSM-IV	Prazosin 14.5 + 3.14 (2–16) vs. placebo	NR
Ramaswamy et al. (2017)	29/30	32.70 (7.10)	57 (97%)	32	12	CAPS: 75.30(14.00)/75.60(12.15)	Veterans	All combat-related	DSM-IV	Vilazodone (10–40) vs. placebo	NR
Raskind et al. (2007)	20/20	26.00 (9.00)	2 (5%)	26	8	CAPS: 76.00 (22.00)/78.00 (18.00)	Veterans	All combat-related	DSM-IV	Prazosin 13.3 + 3 (2–15) vs. placebo	NR
Raskind et al. (2018)	152/152	51.85 (13.78)	297 (98%)	203	10	CAPS: 80.70(15.50)/81.90(17.10)	Veterans	All combat-related	DSM-IV	Prazosin 14.8 + 6.1 (1–20) mg for men or 14.8 + 3.1 (1–12) mg for women vs. placebo	NR
Rezaei Ardani et al. (2017)	12/12	50.22 (5.66)	24 (100%)	NR	12	PCL: 48.00(7.03)/51.00(4.15)	Veterans	All combat-related	DSM-IV-TR	Rivastigmin 3–6 mg/d vs. placebo	NR
Rodgman et al. (2016)	8/7	34.80 (8.30)	8 (53%)	2	2	CAPS: 85.60(45.77)/96.10(57.13); PCL: 43.30(18.97)/31.30(18.47)	Veterans	All combat-related	DSM-IV	Doxazosin 4 mg/d increased by 4 mg every 4 days to 16 mg/d vs. placebo	NR
Rothbaum et al. (2014)	53/50	34.90 (9.10)	98 (95%)	46	6	CAPS: 85.30(27.12)/88.00(20.38); PSS: 32.90(12.07)/32.40(23.27)	Veterans	All combat-related	DSM-IV	D-cycloserine vs. alprazolam	NR
Rothbaum et al. (2014)	53/53	34.9 (9.10)	99	42	6	CAPS: 85.30(27.12)/82.60(17.83); PSS: 32.90(12.07)/32.40(12.2	Veterans	All combat-related	DSM-IV	D-cycloserine vs. placebo	NR
Rothbaum et al. (2014)	50/53	34.9 (9.10)	99 (96%)	43	6	CAPS: 88.00(20.38)/82.60(17.83); PSS: 32.40(23.27)/32.40(12.26)	Veterans	All combat-related	DSM-IV	Alprazolam vs. placebo	NR
Stein et al. (2002)	10/9	53.26 (7.44)	19 (100%)	NR	8	CAPS: 86.10 (22.10)/84.00(16.20)	Veterans	All combat-related	DSM-IV	Olanzapine 15 + 5.25 (10–20) vs. placebo	20.00–25.00
Surís et al. (2017)	26/28	37.5 (14.15)	54 (100%)	36	2	PCL: 56.35 (10.98)/55.39 (10.96)	Veterans	All combat-related	DSM-IV-TR	Dexamethasone 0.15 mg/kg vs. placebo	12.10 (15.00)
Villarreal et al. (2016)	42/38	52.95 (11.07)	75 (94%)	42	12	CAPS: 75.4 (16.00)/70.60(11.70); DTS: 91.38(22.60)/84.47(22.50)	Veterans	All combat-related	DSM-IV	Quetiapine 258 (25–800) vs. placebo	NR
Zohar et al. (2002)	23/19	39.64 (7.56)	37 (88%)	NR	10	CAPS: 91.20(13.30)/93.30(11.70)	Veterans	32 combat-related	DSM-III-R	Sertraline 120 + 60 (50–200) mg/d vs. placebo	7.33 (7.93)

Note: CAPS, Clinician-Administered PTSD Scale; DTS, Davidson Trauma Scale; DSM, Scale for evaluating the symptoms of post-traumatic stress disorder; IES, Impact of Event Scale; I/C, Intervention/control; NR, Not reported; PCL, Post-traumatic stress disorder Checklist; PTSD, Post-traumatic stress disorder; PSS, PTSD Symptom Scale; r, Range.

### Quality Assessment

Thirteen studies showed that the risk of random bias was low, and nine studies indicated that opaque envelopes or placebos with the same appearance as drugs were used in distribution. All studies indicated the use of double blind to carry out the experiment. Thirty-four studies have specific descriptions of patients who drop out. The details and overall risks of bias in the study are shown in Supplementary Figure 1.

Outcome for comparison with placebo.

### Efficacy

#### Change in Total Post-Traumatic Stress Disorder Symptom Scale

**FIGURE 2:**
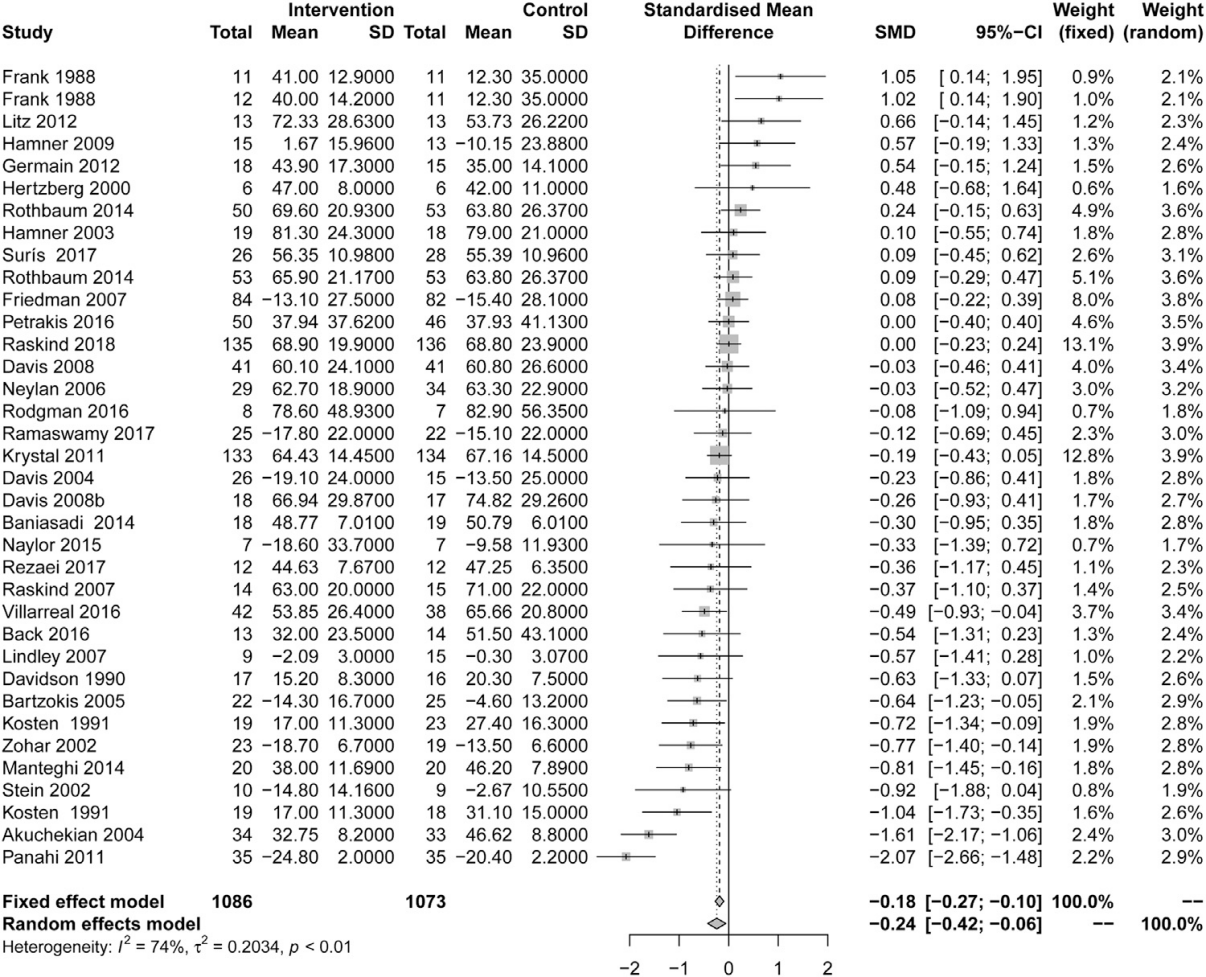
Comparison of active drug compared with placebo for the change in total PTSD symptoms scale.

In comparison with placebo, drug therapy showed a certain therapeutic effect (SMD = −0.24, 95% CI [−0.42, −0.06], I^2^ = 74%) (Figure 2). Among them, the types of drugs, including AASs (SMD = −0.29, 95% CI [−0.48, −0.11], I^2^ = 15%) and central muscle relaxants (SMD = −0.81, 95% CI [−1.45, −0.16], I^2^ = NA), had significant therapeutic effects, but other types of drugs, including alpha blockers, anticonvulsants, corticosteroids, D-Cycloserine, γ-aminobutyric acid (GABA) agonists, MAOIs, N-acetylcysteine, reversible cholinesterase inhibitor, SARIS, serotonin modulators and stimulators (SMSs), SSRIs, TCAs and α2A receptor agonists, had not statistically significant (Table 2).

**TABLE 2:** Comparison of active drug compared with placebo for primary outcomes.

Active drug	Change in total PTSD symptoms scale	Acceptability	Response	Frequencies of complications
AAS				
Risperidone	Three studies Bartzokis et al. (2005); Hamner et al. (2009); Krystal et al. (2011), N = 369, SMD = −0.22, 95% CI [−0.43, 0.00], I^2^ = 32%	Four studies Bartzokis et al. (2005); Hamner et al. (2003); Krystal et al. (2011); Monnelly et al. (2003), N = 384, RR = 1.31, 95% CI [0.82, 2.59], I^2^ = 0%	NR	Three studies Bartzokis et al. (2005); Hamner et al. (2009); Krystal et al. (2011), N = 369, RR = 1.20, 95% CI [0.36, 4.01], I^2^ = 0%
Aripiprazole	One study Naylor et al. (2015), N = 14, SMD = −0.33, 95% CI [−1.39, 0.72], I^2^ = NA	One study Naylor et al. (2015), N = 14, RR = 5.00, 95% CI [0.29, 87.54], I^2^ = NA	NR	One study Naylor et al. (2015), N = 14, RR = 1.00, 95% CI [0.02, 44.12], I^2^ = NA
Olanzapine	One study Stein et al. (2002), N = 19, SMD = −0.92, 95% CI [−1.88, 0.04], I^2^ = NA	One study Stein et al. (2002), N = 19, RR = 1.27, 95% CI [0.32, 4.96], I^2^ = NA	One study Stein et al. (2002), N = 19, RR = 2.70, 95% CI [0.34, 21.53], I^2^ = NA	One study Stein et al. (2002), N = 19, RR = 4.52, 95% CI [0.25, 82.77], I^2^ = NA
Quetiapine	One study Villarreal et al. (2016), N = 80,SMD = −0.49, 95% CI [−0.93, −0.04], I^2^ = NA	One study Villarreal et al. (2016), N = 80, RR = 0.60, 95% CI [0.35, 1.01], I^2^ = NA	NR	One study Villarreal et al. (2016), N = 80, RR = 2.46, 95% CI [0.72, 7.33], I^2^ = NA
Alpha blockers				
Prazosin	Four studies Germain et al. (2012); Petrakis et al. (2016); Raskind et al. (2018); Raskind et al. (2007), N = 429, SMD = 0.02, 95% CI [−0.17, 0.21], I^2^ = 8%	Four studies Germain et al. (2012); Petrakis et al. (2016); Raskind et al. (2018); Raskind et al. (2007), N = 429, RR = 0.88, 95% CI [0.57, 1.35], I^2^ = 17%	One study Raskind et al. (2007), N = 40, RR = 6.00, 95% CI [1.54, 22.34], I^2^ = NA	Three studies Germain et al. (2012); Petrakis et al. (2016); Raskind et al. (2007), N = 169, RR = 1.17, 95% CI [0.47, 2.92], I^2^ = 0%
Doxazosin	One study Rodgman et al. (2016), N = 15, SMD = −0.08, 95% CI [−1.09, 0.94], I^2^ = NA	One study Rodgman et al. (2016), N = 15, RR = 0.88, 95% CI [0.58, 1.34], I^2^ = NA	NR	NR
Anticonvulsants				
Topiramate	Two studies Akuchekian and Amanat (2004); Lindley et al. (2007), N = 91, SMD = −1.14, 95% CI [−2.16, −0.12], I^2^ = 76%	Three studies Akuchekian and Amanat (2004); Batki et al. (2014); Lindley et al. (2007), N = 137, RR = 1.42, 95% CI [0.70, 2.86], I^2^ = 17%	NR	Three studies Akuchekian and Amanat (2004); Batki et al. (2014); Lindley et al. (2007), N = 137, RR = 3.31, 95% CI [1.09, 10.06], I^2^ = 0%
Divalproex	Two studies Davis et al. (2008a); Hamner et al. (2009), N = 110, SMD = 0.19, 95% CI [−0.38, 0.75], I^2^ = 45%	Two studies (Davis et al. (2008a); Hamner et al. (2009), N = 110, RR = 0.87, 95% CI [0.46, 1.62], I^2^ = 0%	NR	Two studies Davis et al. (2008a); Hamner et al. (2009), N = 110, RR = 2.31, 95% CI [0.48, 11.23], I^2^ = 0%
Central muscle relaxant				
Baclofen	One study Manteghi et al. (2014), N = 40, SMD = -0.81, 95% CI [-1.45, -0.16], I^2^ = NA	One study (Manteghi et al. (2014), N = 40, RR = 1.00, 95% CI [0.02, 48.09], I^2^ = NA	NR	NR
Corticosteroid				
Dexamethasone	One study Surís et al. (2017), N = 54, SMD = 0.09, 95% CI [−0.445, 0.62], I^2^ = NA	One study Surís et al. (2017), N = 54, RR = 1.97, 95% CI [0.85, 4.57], I^2^ = NA	NR	NR
D-cycloserine				
D-cycloserine	Two studies Litz et al. (2012); Rothbaum et al. (2014), N = 132, SMD = 0.19, 95% CI [−0.15, 0.54], I^2^ = 38%	Two studies Litz et al. (2012); Rothbaum et al. (2014), N = 132, RR = 1.00, 95% CI [0.06, 15.49], I^2^ = 0%	NR	NR
GABA agonists				
Pregabalin	One study Baniasadi et al. (2014), N = 37, SMD = −0.30, 95% CI [−0.95, 0.35], I^2^ = NA	One study Baniasadi et al. (2014), N = 37, RR = 1.05, 95% CI [0.02, 50.43], I^2^ = NA	NR	NR
Alprazolam	One study Rothbaum et al. (2014), N = 103, SMD = 0.24, 95% CI [−0.15, 0.63], I^2^ = NA	One study Rothbaum et al. (2014), N = 103, RR = 1.06, 95% CI [0.02, 52.39], I^2^ = NA	NR	NR
MAOI				
Phenelzine	Two studies Frank et al. (1988); Kosten et al. (1991), N = 60, SMD = −0.03, 95% CI [−2.05, 1.99], I^2^ = 92%	Two studies Frank et al. (1988); Kosten et al. (1991), N = 60, RR = 0.36, 95% CI [0.16, 0.85], I^2^ = 0%	One study Kosten et al. (1991), N = 37, RR = 2.46, 95% CI [1.10, 5.51], I^2^ = NA	One study Kosten et al. (1991), N = 37, RR = 0.32, 95% CI [0.04, 2.76], I^2^ = NA
N-acetylcysteine				
N-acetylcysteine	One study Back et al. (2016), N = 27, SMD = −0.27, 95% CI [−1.31, 0.23], I^2^ = NA	One study Back et al. (2016), N = 27, RR = 1.49, 95% CI [0.44, 5.60], I^2^ = NA	NR	One study Back et al. (2016), N = 27, RR = 0.95, 95% CI [0.02, 45.26], I^2^ = NA
Reversible cholinesterase inhibitor				
Rivastigmine	One study Rezaei Ardani et al. (2017), N = 24, SMD = −0.36, 95% CI [−1.17, 0.45], I^2^ = NA	One study Rezaei Ardani et al. (2017), N = 24, RR = 1.00, 95% CI [0.02, 46.70], I^2^ = NA	NR	One study Rezaei Ardani et al. (2017), N = 24, RR = 1.00, 95% CI [0.02, 46.70], I^2^ = NA
SARIs				
Nefazodone	One study Davis et al. (2004), N = 41, SMD = −0.23, 95% CI [−0.86, 0.41], I^2^ = NA	One study Davis et al. (2004), N = 41, RR = 1.15, 95% CI [0.55, 2.43], I^2^ = NA	One study Davis et al. (2004), N = 41, RR = 1.04, 95% CI [0.43, 2.53], I^2^ = NA	One study Davis et al. (2004), N = 41, RR = 2.88, 95% CI [0.37, 22.43], I^2^ = NA
SMSs				
Valizodone	One study Ramaswamy et al. (2017), N = 47, SMD = −0.12, 95% CI [−0.69, 0.45], I^2^ = NA	One study Ramaswamy et al., 2017), N = 47, RR = 0.55, 95% CI [0.17, 1.53], I^2^ = NA	NR	One study Ramaswamy et al. (2017), N = 47, RR = 3.10, 95% CI [0.13, 73.13], I^2^ = NA
SSRIs				
Sertraline	Three studies Friedman et al. (2007); Panahi et al. (2011); Zohar et al. (2002), N = 178, SMD = −0.90, 95% CI [−2.22, 0.42], I^2^ = 95%	Three studies Friedman et al. (2007); Panahi et al. (2011); Zohar et al. (2002), N = 178, RR = 1.36, 95% CI [0.85, 2.19], I^2^ = 24%	NR	Three studies Friedman et al. (2007); Panahi et al. (2011); Zohar et al. (2002), N = 178, RR = 2.18, 95% CI [0.93, 5.12], I^2^ = 0%
Fluoxetine	One study Hertzberg et al. (2000), N = 12, SMD = 0.48, 95% CI [−0.68, 1.64], I^2^ = NA	One study Hertzberg et al. (2000), N = 12, RR = 3.00, 95% CI [0.15, 60.88], I^2^ = NA	One study Hertzberg et al. (2000), N = 12, RR = 0.50, 95% CI [0.06, 4.15], I^2^ = NA	One study Hertzberg et al. (2000), N = 12, RR = 3.00, 95% CI [0.15, 60.88], I^2^ = NA
TCAs				
Amitriptyline	One study Davidson et al. (1990), N = 46, SMD = −0.63, 95% CI [−1.33, 0.07], I^2^ = NA	One study Davidson et al. (1990), N = 46, RR = 1.30, 95% CI [0.53, 3.22], I^2^ = NA	One study Davidson et al. (1990), N = 46, RR = 3.00, 95% CI [0.98, 9.14], I^2^ = NA	One study Davidson et al. (1990), N = 46, RR = 5.90, 95% CI [0.32, 108.03], I^2^ = NA
Imipmamine	Two studies Frank et al. (1988); Kosten et al. (1991), N = 59, SMD = −0.02, 95% CI [−2.07, 2.03], I^2^ = 92%	Two studies Frank et al. (1988); Kosten et al. (1991), N = 59, RR = 0.80, 95% CI [0.48, 1.32], I^2^ = 0%	One study Kosten et al. (1991), N = 41, RR = 2.35, 95% CI [1.05, 5.24], I^2^ = NA	One study Kosten et al. (1991), N = 41, RR = 1.01, 95% CI [0.29, 3.56], I^2^ = NA
α2 receptor agonist				
Mirtazapine	Two studies Davis et al. (2008b); Neylan et al. (2006), N = 98, SMD = −0.11, 95% CI [−0.51, 0.29], I^2^ = 0%	Two studies Davis et al. (2008b); Neylan et al. (2006), N = 98, RR = 2.15, 95% CI [0.79, 5.82], I^2^ = 0%	NR	One study Neylan et al. (2006), N = 63, RR = 8.17, 95% CI [0.44, 151.84], I^2^ = NA

Note: AAS, Atypical antipsychotics; GABA agonists, G-aminobutyric acid agonists; MAOI, Monoamine oxidase inhibitor; SARIs, Serotonin antagonist and reuptake inhibitors; SMSs, Serotonin modulator and stimulators; SSRIs, Selective serotonin reuptake inhibitors; TCAs, Tricyclic antidepressants; SMD, Standardized mean difference; RR, Relative risk; CI, Confidence interval; NA, Not applicable; NR, Not reported.

#### Subsymptoms

##### Avoidance

In comparison with placebo, drug therapy has a significant effect on the improvement of avoidance (SMD = −0.26, 95% CI [−0.45, −0.07], I^2^ = 52%). Among them, the types of drugs, including: MAOIs (SMD = −0.67, 95% CI [−1.13, −0.22], I^2^ = 0%) and SSRIs (SMD = −0.67, 95% CI [−1.00, −0.34], I^2^ = NA) had significant therapeutic effects. Other types of drugs, including: AASs, alpha blockers, anticonvulsants, N-acetylcysteine, reversible cholinesterase inhibitor, SARIs, α2A receptor agonists and TCAs had no statistical significance (Supplementary Table 1).

##### Hyper-Arousal

In comparison with placebo, drug therapy has a significant effect on the improvement of hyper−arousal (SMD = −0.31, 95% CI [−0.46, −0.16], I^2^ = 35%). Among them, SSRIs (SMD = −0.72, 95% CI [−1.05, −0.39], I^2^ = 0%) had significant therapeutic effects. Other types of drugs, including AASs, alpha blockers, anticonvulsants, N-acetylcysteine, reversible cholinesterase inhibitor, SARIs and α2A receptor agonists had no statistically significant results (Supplementary Table 1).

##### Re-experiencing

In comparison with placebo, drug therapy has a significant effect on the improvement of re−experiencing (SMD = −0.30, 95% CI [−0.42, −0.18], I^2^ = 39%). Among them, AASs (SMD = −0.38, 95% CI [−0.57, −0.19], I^2^ = 0%) and MAOIs (SMD = −0.83, 95% CI [−1.37, −0.37], I^2^ = 0%) had significant therapeutic effects. Other types of drugs, including alpha blockers, anticonvulsants, N-Acetylcysteine, reversible cholinesterase inhibitors, SARIs, SSRIs, TCAs and α2A receptor agonists had no statistically significant results (Supplementary Table 1).

#### Response

**FIGURE 3:**
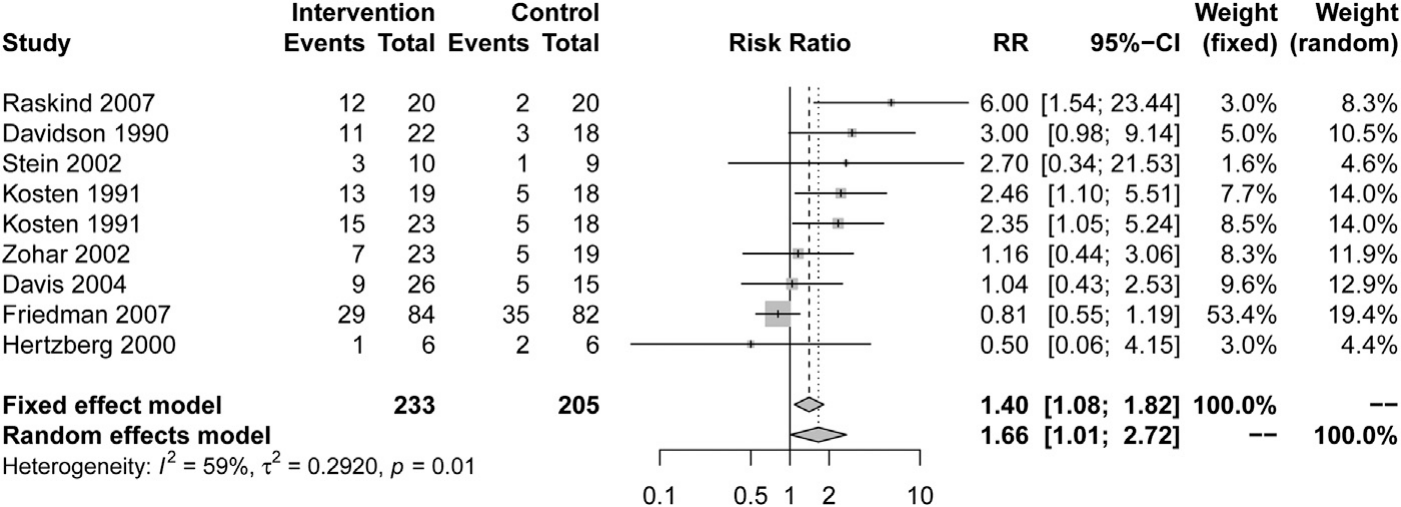
Comparison of active drug compared with placebo for the response.

Patients who received drug treatment had higher response rates than placebo (RR = 1.66, 95% CI [1.01, 2.72], I^2^ = 59%) (Figure 3). Alpha blockers (RR = 6.00, 95% CI [1.54, 23.44], I^2^ = NA), MAOI (RR = 2.46, 95% CI [1.10, 5.51], I^2^ = NA) and TCAs (RR = 2.59, 95% CI [1.35, 4.98], I^2^ = 0%) showed a higher response rate. Other types of drugs, including AASs, SARIs and SSRIs, had no statistical significance in Table 2.

#### Outcomes for Active Drug Compared with Active Comparators

In comparison with other drugs, α2 receptor agonist (mirtazapine) contrast SSRIs (sertraline) from (Chung et al., 2004) change in total PTSD symptoms scale: (SMD = −0.57, 95% CI [−0.97, 0.17], I^2^ = NA), acceptability: (RR = 1.12, 95% CI [0.41, 3.10], I^2^ = NA), response: (RR = 1.04, 95% CI [0.82, 1.32], I^2^ = NA), frequencies of complications: (RR = 1.25, 95% CI [0.60, 2.58], I^2^ = NA). D−Cycloserine contrast GABA agonists change in total PTSD symptoms scale: (alprazolam) from (Rothbaum et al., 2014) (SMD = −0.17, 95% CI [−0.56, 0.21], I^2^ = NA), acceptability: (RR = 0.94, 95% CI [0.02, 46.68], I^2^ = NA). TCAs (imipramine) contrast MAOIs (phenelzine) from (Frank et al., 1988; Kosten et al., 1991) change in total PTSD symptoms scale: (SMD = 0.17, 95% CI [−0.33, 0.66], I^2^ = 0%), frequencies of complications: (RR = 0.30, 95% CI [0.04, 2.48], I^2^ = NA), acceptability: (RR = 0.45, 95% CI [0.19, 1.09], I^2^ = 0%) in Table 3.

**TABLE 3:** Comparison between active drugs for primary outcomes.

Intervention	Control	SMD/RR	95% CI	P for SMD/RR	I^2^
Change in total PTSD symptoms scale					
D-cycloserine	Alprazolam	−0.17	[−0.56, 0.20]	0.26	NA
Imipmamine	Phenelzine	0.17	[−0.33, 0.66]	0.32	0%
Mirtazapine	Sertraline	−0.57	[−0.97, −0.17]	<0.05	NA
Accept					
D-cycloserine	Alprazolam	0.94	[0.02, 46.71]	0.92	NA
Imipmamine	Phenelzine	0.45	[0.19, 1.09]	0.36	NA
Mirtazapine	Sertraline	1.12	[0.41, 3.10]	0.49	NA
Response					
Mirtazapine	Sertraline	1.04	[0.82, 1.32]	0.30	NA
Frequencies of complications					
Imipmamine	Phenelzine	0.30	[0.04, 2.48]	0.56	NA
Mirtazapine	Sertraline	1.25	[0.60, 2.58]	0.54	NA

Note: SMD, Standardized mean difference; RR, Relative risk; CI, Confidence interval; NA, Not applicable; NR, Not reported; PTSD, Post-traumatic stress disorder.

### Frequencies of Complications

**FIGURE 4:**
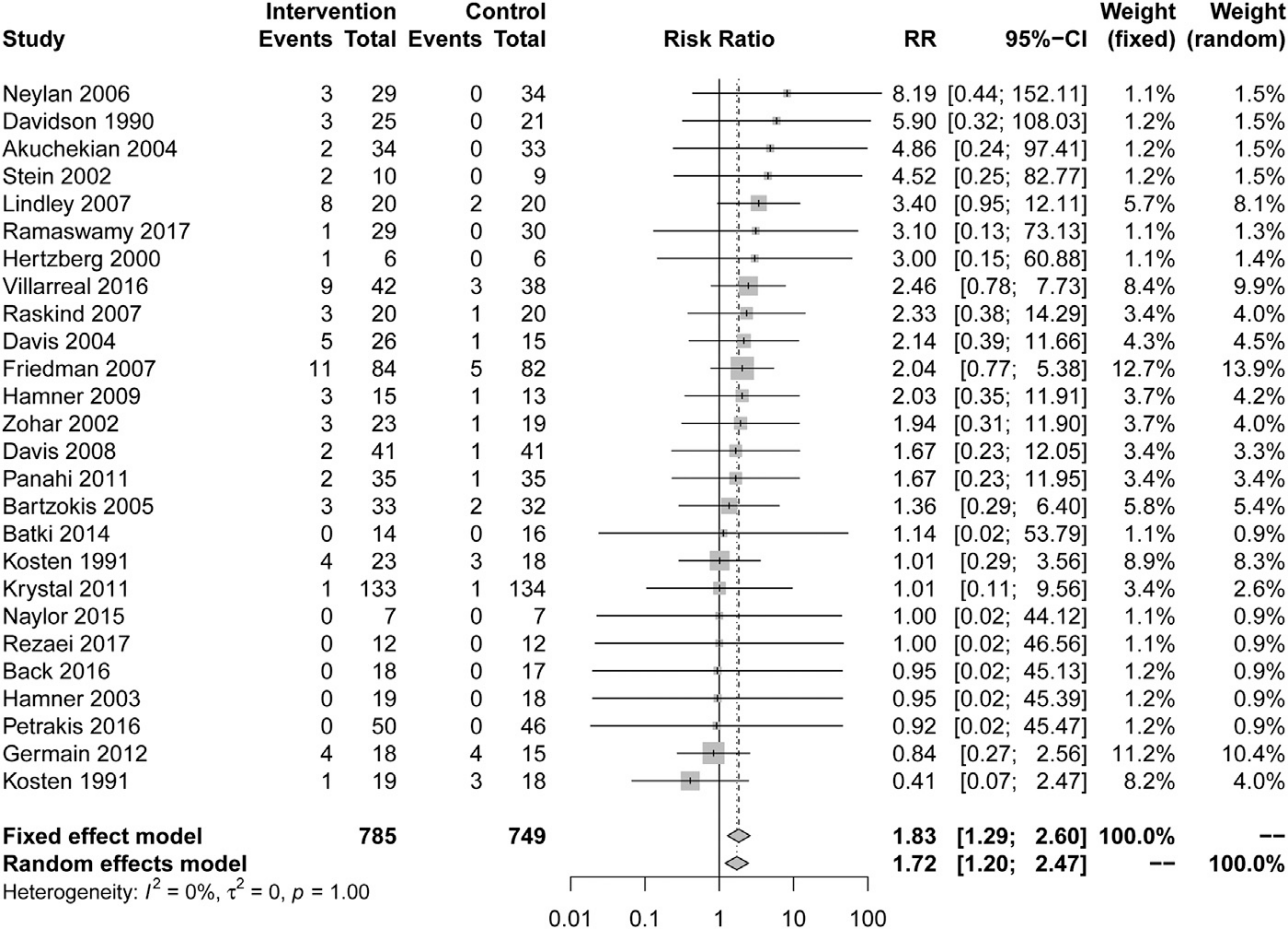
Comparison of active drug compared with placebo for the frequencies of complications.

In comparison with placebo, there are more complications caused by drug treatment. (RR = 1.83, 95% CI [1.29, 2.60], I^2^ = 0%) in Figure 4. All types of drugs showed no positive effect compared to placebo. Among them, anticonvulsants (RR = 2.62, 95% CI [1.13, 6.09], I^2^ = 0%) showed a higher risk of complications. Other types of drugs, including AASs, alpha blockers, MAOI, N-acetylcysteine, reversible cholinesterase inhibitor, SARIs, SMSs, SSRIs, TCAs and α2A receptor agonists showed no statistical significance compared with placebo in Table 2.

### Acceptability

**FIGURE 5:**
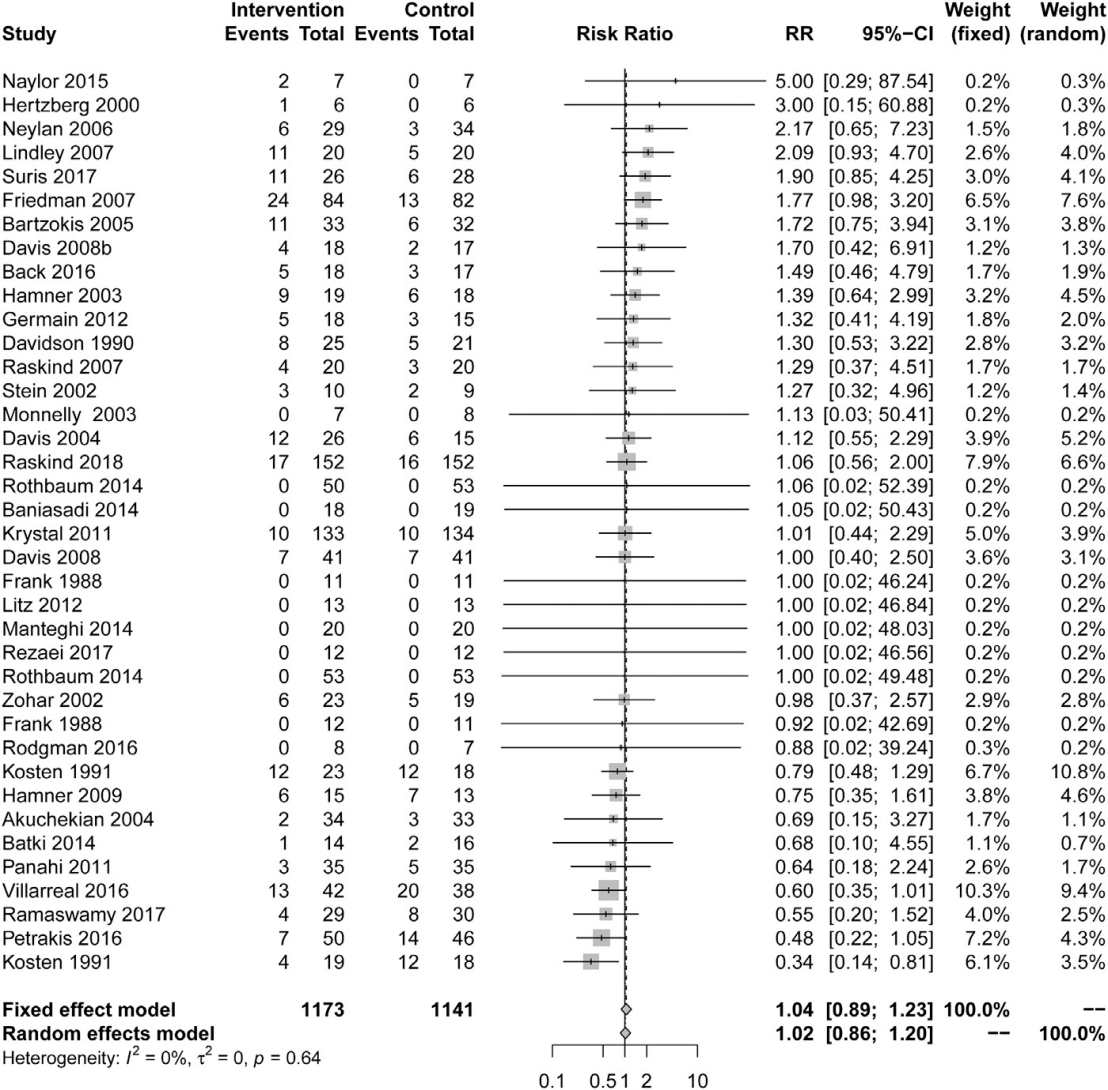
Comparison of active drug compared with placebo for the acceptability.

In comparison with placebo, more people were withdrawn from the study due to drug treatment, but it was not statistically significant (RR = 1.04, 95% CI [0.89, 1.23], I^2^ = 0%) in Figure 5. Only one drug type showed a lower withdrawal rate than a placebo: MAOIs (RR = 0.36, 95% CI [0.16, 0.85], I^2^ = 0%), and other types of drugs, including AASs, alpha blockers, anticonvulsant, central muscle relaxants, corticosteroid, D-Cycloserine, GABA agonists, N-acetylcysteine, reversible cholinesterase inhibitors, SMSs, SARIs, SSRIs, TCAs and α2A receptor agonists had no statistical significance in Table 2.

### Stratified Analyses

#### The Efficacy of Drugs in the Treatment of Patients with Comorbidities

For stratified analyses with comorbidity in comparison with placebo, the outcomes, including change in total PTSD symptom scale with comorbidity (SMD = −0.16, 95% CI [−0.29, −0.04], I^2^ = 29%) and symptoms of re−experiencing (SMD = −0.25, 95% CI [−0.43, −0.07], I^2^ = 27%), had a statistical significance, but other outcomes, including symptoms of hyper-arousal, symptoms of avoidance, acceptability, response and frequencies of complications, had not statistical significance in Supplementary Table 2.

For the patients without comorbidity in comparison with placebo, symptoms of avoidance (SMD = −0.31, 95% CI [−0.54, −0.08], I^2^ = 53%), symptoms of hyper−arousal (SMD = −0.31, 95% CI [−0.49, −0.14], I^2^ = 39%), symptoms of re−experiencing (SMD = −0.36, 95% CI [−0.52, −0.20], I^2^ = 39%) and frequencies of complications (RR = 1.68, 95% CI [1.11, 2.56], I^2^ = 0%), had a statistical significance, other outcomes, including change in total PTSD symptom scale, acceptability and response, had no statistical significance in Supplementary Table 2.

#### Effect of Previous Treatment on Drug Treatment

Aim at patients with previous medication in comparison with placebo, Supplementary Table 3 showed these outcomes, including change in total PTSD symptom scale (SMD = −0.05, 95% CI [−0.35, 0.25], I^2^ = 74%), response (RR = 4.86, 95% CI [1.58, 14.99], I^2^ = 0%) and frequencies of complications (RR = 2.59, 95% CI [1.26, 5.33], I^2^ = 0%), were statistically different, but other outcomes, including symptoms of avoidance, symptoms of hyper-arousal, symptoms of re-experiencing and acceptability, had no statistical significance in Supplementary Table 3.

Aim at patients without previous medication in comparison with placebo, Supplementary Table 3 showed these outcomes, including change in total PTSD symptom scale (SMD = −0.32, 95% CI [−0.53, −0.12], I^2^ = 72%), symptoms of avoidance (SMD = −0.34, 95% CI [−0.54, −0.13], I^2^ = 45%), symptoms of hyper−arousal (SMD = −0.46, 95% CI [−0.68, −0.25], I^2^ = 23%), symptoms of re−experiencing (SMD = −0.39, 95% CI [−0.53, −0.25], I^2^ = 34%) and frequencies of complications (RR = 1.48, 95% CI [1.03, 2.13], I^2^ = 0%), were statistically different, but other outcomes, including acceptability and response, had no statistical significance.

#### Gender

For males veterans, Supplementary Table 4 demonstrated that the outcomes, including symptoms of avoidance (SMD = −0.52, 95% CI [−0.78, −0.25], I^2^ = 0%), symptoms of hyper−arousal (SMD = −0.76, 95% CI [−1.06, −0.46], I^2^ = 0%) and symptoms of re−experiencing (SMD = −0.53, 95% CI [−0.77, −0.28], I^2^ = 0%), had a statistically different. However, the other outcomes, including change in total PTSD symptom scale, acceptability, response and frequencies of complications, had no statistically different. No studies had been specialized reported on female veterans.

#### Battlefield

In Operation Enduring Freedom and Operation Iraqi Freedom (OEF&OIF), our result in Supplementary Table 5 showed that the outcomes, including symptoms of avoidance (SMD = −0.76, 95% CI [−1.15, −0.37], I^2^ = 0%), symptoms of hyper−arousal (SMD = −0.83, 95% CI [−1.22, −0.44], I^2^ = 0%) and symptoms of re−experiencing (SMD = −0.56, 95% CI [−1.02, −0.10], I^2^ = 0%), were statistically different, but other outcomes, including change in total PTSD symptoms scale, acceptability and frequencies of complications had not.

In vietnam war, our result in Supplementary Table 5 showed that the outcomes, including symptoms of avoidance (SMD = −0.31, 95% CI [−0.59, −0.04], I^2^ = 30%) and symptoms of re−experiencing (SMD = −0.48, 95%CI [−0.77, −0.20], I^2^ = 18%), were statistically different, but other outcomes, including change in total PTSD symptoms scale, symptoms of hyper-arousal, acceptability, response and frequencies of complications, had no statistically different.

#### Different Receptor

Among the drugs acting on 5-HT receptors, our results showed that symptoms of hyper-arousal (SMD = −0.54, 95% CI [−0.86, −0.21], I^2^ = 0%), symptoms of re−experiencing (SMD = −0.62, 95% CI [−0.86, −0.39], I^2^ = 24%) and symptoms of avoidance (SMD = −0.53, 95% CI [− 0.77, −0.3], I^2^ = 0%), were statistically different, but other outcomes, including change in total PTSD symptoms scale, response, acceptability and frequencies of complications, had no statistically different.

The drugs acting on GABA receptor showed higher complication rate than placebo (RR = 2.64, 95% CI [1.05, 6.65], I^2^ = 0%). The drugs acting on dopamine receptors, our results showed that symptoms of re−experiencing (SMD = −0.35, 95% CI [−0.55, −0.16], I^2^ = 30%) was statistically different. The drugs acting on α1 receptor showed higher response rate than placebo (RR = 6, 95% CI [1.54, 23.44], I^2^ = NA). The drugs acting on α2 receptor has a significant effect on reducing total PTSD symptoms scale (SMD = −0.34, 95% CI [−0.62, −0.06], I^2^ = 31%). All the results of drugs acting on other receptors were not statistically significant in Supplementary Tables 6,7.

### Publication Bias

To analyze whether there is publication bias, we made a funnel chart of the outcomes of more than 10 studies (Supplementary Figure 2A-G).

## Discussion

A total of 36 RCT patients with 2,331 patients were included in this study to evaluate the efficacy of drugs as a whole and various types of drugs in the treatment of veterans. This result showed that three major categories of drugs, including AAS, central muscle relaxant, phenelzine, were effective for veterans with PTSD symptoms in which the risperidone and sertraline showed more effective.

In terms of the comparative efficacy of drugs and placebo, the overall scale effect showed that drug treatment was more effective. This is similar to the recent meta-analysis of adult PTSD (Hoskins et al., 2015; Puetz et al., 2015). Among the many drugs, only AAS, central muscle relaxant and phenelzine are effective. There was no significant difference between other kinds of drugs, and there was only one study on relaxants (Manteghi et al., 2014) or phenelzine (Kosten et al., 1991) for overall scale effect. Unlike other adult PTSD studies that suggest SSRIs are more effective (Stein et al., 2006; Koirala et al., 2017; Huang et al., 2020), in this study, AASs are the only effective drug with a relatively good reputation. The drugs acting on α2 receptor showed a better effect on the overall PTSD score. Psychophysiological studies hadshown that the sympathetic system of PTSD patients in veterans is enhanced (Park et al., 2017), The other study had confirmed the effect of norepinephrine on PTSD (Kosten et al., 1987). However, the drugs acting on α1 receptor do not had a beneficial effect, which may be due to the fact that α1 receptor has little effect on the release of synapses and transmitters. In terms of scale effect values, men had a more positive response to medication, but not statistically significant in terms of response rate. PTSD is usually accompanied by comorbid psychosis, such as alcohol use disorder (AUD) and depression, which may lead to higher frequencies of psychological problems (Blanco et al., 2013). However, the APA guidelines did not find that there were difference in the significant efficacy of active drug for the patient with comorbid (Association, 2017). In our study, the efficacy of drugs in the treatment of patients with comorbid psychosis was better than that of patients with single PTSD. This may indicate that in the case of comorbid mental illness, the use of medication is indispensable.

In the three symptom assessments, drug treatment also showed a better therapeutic effect. However, there are few types of drugs that work. Among them, phenelzine and sertraline had a good effect on the two symptoms, which was similar to the conclusion of the previous meta-analysis (Huang et al., 2020). On the other hand, AASs only had good effects on re-experiencing. Three of these studies (Bartzokis et al., 2005; Hamner et al., 2009; Krystal et al., 2011) used risperidone as an intervention drug, which is sufficient to demonstrate the effectiveness of risperidone. On the other hand, drug therapy is more effective in patients without comorbid psychosis, in which sertraline has a good effect. Moreover, the curative effect of a single drug is better than that of mixed therapy. In the effect of subsymptoms, both 5-HT receptors and dopamine receptors showed better effects. Recent studies had shown that 5-HT has a therapeutic effect on subsymptoms of PTSD, but it is similar to placebo in terms of overall symptoms (Spangler et al., 2020). On the other hand, the drugs acting on dopamine receptors had a relatively good prospect (Scheggia et al., 2018), it is hoped that there will be more drugs to study dopamine receptors in the future.

In terms of the overall response rate, drug therapy is effective, which is the same conclusion reached by the previous meta-analysis (Gu et al., 2016). The effective drugs are MAOIs, alpha blockers and TCAs. There was only one study with small samples on phenelzine (Kosten et al., 1991) or prazosin (Raskind et al., 2007), and two studies (Davidson et al., 1990; Kosten et al., 1991) on TCAs, including imipramine and amitriptyline. Drugs acting on α1 receptor have better response rates, but the number and sample of each type of research is very small. Therefore, we had reservations about effective drugs with response rates, and this result may need to be confirmed by more research.

Although the overall effect of drug treatment is better than that of placebo, in terms of the frequencies of complications, the frequencies of drugs was significantly higher than that of placebo. Among them, all drugs except SARIs and SSRIs showed a higher frequencie of complications, and anticonvulsant drugs have the most obvious increase in frequencies. Our research shows that topiramate and divalproex significantly increase the frequencies of complications in veterans, and also show drugs that act on GABA receptors may lead to a higher frequencies of complications but different opinions had been put forward from the APA guidelines (Association, 2017). The reason for the increase in the frequencies of complications may be that most of the studies using anticonvulsants drugs are treated with mixed drugs. Mechanistically, the action of topiramate on GABA receptors may aid in suppressing the fear response, which is helpful to relieve the symptoms of PTSD (Sofuoglu et al., 2014). Comorbid psychosis is not excluded, drugs conflict with each other, or the right medicine is not prescribed.

There have certain advantages in the withdrawal rate of overall drug therapy, but no advantage was observed in the terms of gender difference, co-disease difference, different receptors and intervention difference. It may prevent us from making effective and accurate assessments.

From the point of view between active comparators, a study (Chung et al., 2004) showed that the scale reduction score and response rate of mirtazapine are better than those of sertraline, but the withdrawal rate and the response were also higher in mirtazapine. Relevant study (McGuire et al., 2017) has promptted that D-Cycloserine can enhance the therapeutic effect and respond to and remission. In this study, we tried to compare D-Cycloserine with alprazolam. From the reduced score of the scale, D-Cycloserine is better, and in terms of frequencies of complications, alprazolam is better, but there is no statistical significance between the two groups. The final comparison is that imipramine compares with phenelzine. In terms of effectiveness, phenelzine is better, and in terms of security, imipramine is more dominant, there is no statistical significance in the comparison between the two groups. There is less evidence of direct comparison between drugs in our study, it is difficult to prove that a drug has a more positive effect, and more evidence needs to be collected in the future to come to a conclusion.

### Implications for Practice

The APA guidelines from 2017 (Association, 2017) indicate that, fluoxetine, paroxetine, sertraline and venlafaxine are effective in adult PTSD patients and that there is insufficient evidence in the systematic review that a particular drug treatment can have a stronger or weaker effect on veterans. In our inclusion studies, paroxetine and venlafaxine is not involved, and there was only one study on fluoxetine (Hertzberg et al., 2000). Contrary to the guidelines, this study showed that venlafaxine performed worse in response rates and reducing scale scores and will result in higher frequencies of complications and withdrawal rate. With regard to sertraline, our further analysis showed that it was not much different from the effectiveness of placebo but increased the risk of complications. This may indicate that the difference between veterans' PTSD and adult PTSD cannot be ignored. In addition to venlafaxine and sertraline, the NICE guidelines also recommend the use of risperidone to manage PTSD symptoms. Four of our included studies used risperidone as an intervention, of which 2 studies were single drug therapy (Bartzokis et al., 2005; Krystal et al., 2011). From the stratified analysis, the therapeutic effect of single drug was better than that of combined drug, the drugs acting on 5-HT receptor and dopamine receptor have significant effect on PTSD subsymptoms. In our in-depth analysis, although no research has report the response rate of risperidone, but the effective and tolerance of risperidone were proved by this study. Therefore, our results recommended that risperidone should be regarded as the first choice for drug treatment in veterans. Different from the effect of SSRIs on 5-HT receptor (Loonen & Ivanova, 2016), AAS is mainly concentrated in dopamine-2 (D) (Nakamura & Nagamine, 2019), therefore, dopamine-2 (D) may develop as an important research direction.

Unlike fluoxetine, paroxetine, sertraline and venlafaxine recommended in APA and NICE guidelines (Health, 2005; Association, 2017) or previous study (Berger et al., 2007), this study recommended that the risperidone is used as the first-line treatment for veterans. The AAS not only has excellent therapeutic effect, but also has few side effects. Only study (Cheng et al., 2019) showed that AAS can also reduce the risk of hearing problems. When the effect of AAS is not obvious, SSRIs are recommended. In the future, clinical researchers can conduct more studies on MAOIs or phenelzine to prove their effectiveness. In terms of drug receptors, α2 receptor, dopamine receptor and 5-HT receptors has certain curative effect. It's worth noting that the drugs acting on α2 receptor with small sample size need more research. 5-HT receptors are mainly receptors of SSRI, SARI drugs, and its efficacy has also been confirmed by other studies (Kosten et al., 1991; Zohar et al., 2002; Panahi et al., 2011). The drugs of dopamine receptors are mainly risperidone, recent studies had shown that the interaction between antipsychotic drugs and abnormal binding proteins in patients is mediated by dopamine receptors. This newly discovered pharmacodynamic mechanism suggests ways to improve the use of antipsychotic drugs (Scheggia et al., 2018). The other study had shown that genetic information may affect dopamine, and individual differences may also be the cause of unstable drug efficacy (Leggio et al., 2019). At the genetic level, studies have shown that ANKRD55 on chromosome five and ZNF626 on chromosome 19 may be effective in the treatment of PTSD in veterans (Stein et al., 2016), In the future, it may be possible to carry out personalized therapy based on genes. It has been proved by studies (Kang et al., 2003; Hoge et al., 2006), different battlefields may lead to different symptoms of PTSD. However, our analysis includes only two battlefields of OEF & OIF and Vietnam War, and there is no statistically significant difference in each primary outcome between the two battlefields. Therefore, drug therapy may not be effective for soldiers who have served in Vietnam and OEF & OIF, It is hoped that there will be more research on different battlefields in the future. Even if the drug treatment is effective, but according to the standard, the moderate effect size is an SMD range of 0.5–0.8, so the drugs effect is very small (Cohen, 2013), and no drugs show statistically significant high efficacy. Therefore, compared with psychotherapy (Belsher et al., 2019; Rasmussen et al., 2019), drug therapy is more suitable as an adjuvant therapy. It is hoped that future studies can further confirm the results of this study and analyze the differences among different groups in more detail. We also hope to be able to compare the performance-to-price ratio of drug therapy and psychotherapy so as to find the most suitable treatment for patients.

### Advantages and Limitations

The study updated the latest and most comprehensive evidence of drug treatment for veterans and pointed out the positive effects of some drugs, which provided ideas and directions for future clinical trial research and made it possible to improve various guidelines. However, this study also has some limitations. First, there are too few studies on various types of drugs in the subsymptomatic outcomes, which may lead to unstable results, so more research is needed to prove it in the future. Secondly, there lacked direct comparison evidences, such as the special drugs and doses, leading that the corresponding dosages failed recommended for the veterans based on the available evidence. It is expected that there will be more high-quality evidence with large sample in the future.

## Conclusion

This study demonstrated that AASs including risperidone, and SSRIs including sertraline, can effectively reduce the veteran’s score of PTSD scale and its subsymptoms, and the combination of drugs will not improve the therapeutic effect. For veterans with comorbid mental illness, the effect of AASs is more significant, and the effects of anticonvulsants are better for male veterans. However, it is noteworthy that anticonvulsant drugs can lead to more complications. The effect of central muscle relaxants and phenelzine exceeds the medium standard, but more researches are needed to confirm it. Drugs acting on 5-HT receptors and dopamine can significantly improve subsymptoms, while drugs acting on α2 receptors need to be confirmed by more studies. Meanwhile, other drugs including TCAs, SARIs, SMSs, α2 receptor agonist, reversible cholinesterase inhibitor, N-acetylcysteine, MAOI, GABA agonists, D-cycloserine, corticosteroid, alpha blockers may have lower priority.

## Data Availability Statement

The original contributions presented in the study are included in the article/Supplementary Material, further inquiries can be directed to the corresponding authors.

## Author Contributions

Conceptualization: CZ, R-XY; Data curation: Y-FZ, Z-DH; Formal analysis: Y-FZ, Z-DH, H-YG, Funding acquisition: G-LG; Investigation: G-LG, R-XY, CZ; Methodology: YFZ, Z-DH, R-XY, CZ; Project administration: Z-DH, R-XY, CZ; Resources: Z-DH, CZ; Software: Y-FZ, Z-DH; Supervision: G-LG, R-XY, CZ; Validation: R-XY, CZ; Visualization: G-LG, R-XY, CZ; Roles/Writing–original draft: Y-FZ, Z-DH; Writing–review & editing: G-LG, R-XY, CZ.

## Conflict of Interest

The authors declare that the research was conducted in the absence of any commercial or financial relationships that could be construed as a potential conflict of interest.
