# Supplementary material for: Key Clinical Interest Outcomes of Pharmaceutical Administration for Veterans With Post-Traumatic Stress Disorder Based on Pooled Evidences of 36 Randomised Controlled Trials With 2,331 Adults
Source: Front Pharmacol. 2020 Dec 17;11:602447. doi: 10.3389/fphar.2020.602447 (PMC7773915; doi:10.3389/fphar.2020.602447)
Supplement: Supplementary file 1 [file datasheet1.docx]

**Legends of Supplementary Materials**

**Supplementary Method 1** Search strategy

**Supplementary Table 1** Subgroup analysis of different drugs for subsymptoms

**Supplementary Table 2** Subgroup analysis of comorbidities in all outcomes

**Supplementary Table 3** Subgroup analysis of previous treatment in all outcomes

**Supplementary Table 4** Subgroup analysis of pure male in all outcomes

**Supplementary Table 5** Subgroup analysis of different battlefields in all outcomes

**Supplementary Table 6** Subgroup analysis of different acting receptors in primary outcomes

**Supplementary Table 7** Subgroup analysis of different acting receptors in secondary outcomes

**Supplementary Figure 1** Risk of bias summary

**Supplementary Figure 2** Funnel of all outcomes

**Supplementary Method 1** Search strategy

**1. Ovid MEDLINE(R) and Epub Ahead of Print, In-Process & Other Non-Indexed Citations, Daily, and Versions(R)** **<1946 to January 1, 2020>**

1. (Stress Disorders, Post-Traumatic).mp.
2. (post-traumatic stress disorder).mp.
3. (post-traumatic stress disorders).mp.
4. (disorder* AND post-traumatic).ti,ab.
5. (Stress Disorders, Traumatic).mp.
6. (Combat Disorders).mp.
7. PTSD.mp.
8. Veteran.mp.
9. Warfare.mp.
10. (Armed Conflicts).mp.
11. War.ti,ab.
12. #1 OR #2 OR #3 OR #4 OR #5 OR #6 OR #7 OR #8 OR #9 OR #10 OR #11
13. Benzodiazepines.mp.
14. (Antidepressive Agents, Tricyclic).mp.
15. Anticonvulsants.mp.
16. (Adrenergic alpha-Antagonists).mp.
17. (Antipsychotic Agents).mp.
18. (Antidepressive Agents).mp.
19. (citalopram OR escitalopram OR fluoxetine OR fluvoxamine OR paroxetine OR sertraline OR desvenlafaxine OR venlafaxine OR duloxetine OR imipramine OR amitriptyline OR desipramine OR bupropion OR mirtazapine OR nefazodone OR trazodone OR prazosin OR olanzapine OR risperidone OR benzodiazepines OR alprazolam OR diazepam OR lorazepam OR clonazepam OR topiramate OR tiagabine OR lamotrigine OR carbamazepine OR divalproex).mp.
20. #13 OR #14 OR #15 OR #16 OR #17 OR #18 OR #19
21. #12 AND #20/ (1943)

**2. Ovid EMbase <1946 to January 1, 2020>**

1. (Stress Disorders, Post-Traumatic).mp.
2. (post-traumatic stress disorder).mp.
3. (post-traumatic stress disorders).mp.
4. (disorder* AND post-traumatic).ti,ab.
5. (Stress Disorders, Traumatic).mp.
6. (Combat Disorders).mp.
7. PTSD.mp.
8. Veteran.mp.
9. Warfare.mp.
10. (Armed Conflicts).mp.
11. War.ti,ab.
12. #1 OR #2 OR #3 OR #4 OR #5 OR #6 OR #7 OR #8 OR #9 OR #10 OR #11
13. Benzodiazepines.mp.
14. (Antidepressive Agents, Tricyclic).mp.
15. Anticonvulsants.mp.
16. (Adrenergic alpha-Antagonists).mp.
17. (Antipsychotic Agents).mp.
18. (Antidepressive Agents).mp.
19. (citalopram OR escitalopram OR fluoxetine OR fluvoxamine OR paroxetine OR sertraline OR desvenlafaxine OR venlafaxine OR duloxetine OR imipramine OR amitriptyline OR desipramine OR bupropion OR mirtazapine OR nefazodone OR trazodone OR prazosin OR olanzapine OR risperidone OR benzodiazepines OR alprazolam OR diazepam OR lorazepam OR clonazepam OR topiramate OR tiagabine OR lamotrigine OR carbamazepine OR divalproex).mp.
20. #13 OR #14 OR #15 OR #16 OR #17 OR #18 OR #19
21. #12 AND #20/ (3234)

**3. CENTRAL, The** **Cochrane Library < June 2020>**

1. MeSH descriptor: [Stress Disorders, Post-Traumatic] explode all trees
2. (post-traumatic stress disorder):ti,ab,kw
3. (post-traumatic stress disorders):ti,ab,kw
4. (disorder* AND post-traumatic):ti,ab,kw
5. MeSH descriptor: [Stress Disorders, Traumatic] explode all trees
6. MeSH descriptor: [Combat Disorders] explode all trees
7. (PTSD):ti,ab,kw
8. MeSH descriptor: [Veteran] explode all trees
9. MeSH descriptor: [Warfare] explode all trees
10. MeSH descriptor: [Armed Conflicts] explode all trees
11. War:ti,ab,kw
12. #1 OR #2 OR #3 OR #4 OR #5 OR #6 OR #7 OR #8 OR #9 OR #10 OR #11
13. MeSH descriptor: [Benzodiazepines] explode all trees
14. MeSH descriptor: [Antidepressive Agents, Tricyclic] explode all trees
15. MeSH descriptor: [Anticonvulsants] explode all trees
16. MeSH descriptor: [Adrenergic alpha-Antagonists] explode all trees
17. MeSH descriptor: [Antipsychotic Agents] explode all trees
18. MeSH descriptor: [Antidepressive Agents] explode all trees
19. (citalopram OR escitalopram OR fluoxetine OR fluvoxamine OR paroxetine OR sertraline OR desvenlafaxine OR venlafaxine OR duloxetine OR imipramine OR amitriptyline OR desipramine OR bupropion OR mirtazapine OR nefazodone OR trazodone OR prazosin OR olanzapine OR risperidone OR benzodiazepines OR alprazolam OR diazepam OR lorazepam OR clonazepam OR topiramate OR tiagabine OR lamotrigine OR carbamazepine OR divalproex):ti,ab,kw
20. #13 OR #14 OR #15 OR #16 OR #17 OR #18 OR #19
21. #12 AND #20/ (595)

**4. Web of Science (ISI) <1970 to January 1, 2020>**

1. Topic: (PTSD)

Databases= SCI-EXPANDED, SSCI, A&HCI, CPCI-S, CPCI-SSH, BKCI-S, BKCI-SSH, ESCI, CCR-EXPANDED, IC Timespan= 1970-2019

1. Topic: (posttraumatic)

Databases= SCI-EXPANDED, SSCI, A&HCI, CPCI-S, CPCI-SSH, BKCI-S, BKCI-SSH, ESCI, CCR-EXPANDED, IC Timespan= 1970-2019

1. Topic: (post trauma)

Databases= SCI-EXPANDED, SSCI, A&HCI, CPCI-S, CPCI-SSH, BKCI-S, BKCI-SSH, ESCI, CCR-EXPANDED, IC Timespan= 1970-2019

1. Topic: (Veteran)

Databases= SCI-EXPANDED, SSCI, A&HCI, CPCI-S, CPCI-SSH, BKCI-S, BKCI-SSH, ESCI, CCR-EXPANDED, IC Timespan= 1970-2019

1. Topic: (Armed Conflicts)

Databases= SCI-EXPANDED, SSCI, A&HCI, CPCI-S, CPCI-SSH, BKCI-S, BKCI-SSH, ESCI, CCR-EXPANDED, IC Timespan= 1970-2019

1. #5 OR #4 OR #3 OR #2 OR #1

Databases= SCI-EXPANDED, SSCI, A&HCI, CPCI-S, CPCI-SSH, BKCI-S, BKCI-SSH, ESCI, CCR-EXPANDED, IC Timespan= 1970-2019

1. Topic: (pharmacotherapy)

Databases= SCI-EXPANDED, SSCI, A&HCI, CPCI-S, CPCI-SSH, BKCI-S, BKCI-SSH, ESCI, CCR-EXPANDED, IC Timespan= 1970-2019

1. #6 AND #7/ (947)

Databases= SCI-EXPANDED, SSCI, A&HCI, CPCI-S, CPCI-SSH, BKCI-S, BKCI-SSH, ESCI, CCR-EXPANDED, IC Timespan=1970-2019

**Supplementary Table 1** Subgroup analysis of different drugs for subsymptoms

| **Active drug** | **Placebo** | **Avoidance** | **Hyper-arousal** | **Re-experiencing** |
| --- | --- | --- | --- | --- |
| **Active drug VS. Placebo** | | | | |
| **AAS** | | | | |
| Risperidone | | Three studies, N=351, SMD=-0.11, 95%CI[-0.10, 0.32], I^2^=0% | Two studies, N=84, SMD=-0.31, 95%CI[-1.36, 0.74], I^2^=82% | Three studies, N=351, SMD=-0.35, 95%CI[-0.56, -0.14], I^2^=0% |
| Quetiapine | | One study, N=80, SMD=-0.24, 95%CI[-0.68, 0.20], I^2^=NA | One study, N=80, SMD=-0.55, 95%CI[-1.00, -0.10], I^2^=NA | One study, N=80, SMD=-0.53, 95%CI[-0.98, -0.09], I^2^=NA |
| **Alpha blockers** | | | | |
| Prazosin | | One study, N=96, SMD=0.09, 95%CI[-0.31, 0.49], I^2^=NA | One study, N=96, SMD=0.06, 95%CI[-0.34, 0.46], I^2^=NA | One study, N=96, SMD=0.05, 95%CI[-0.35, 0.45], I^2^=NA |
| Doxazosin | | One study, N=15, SMD=0.23, 95%CI[-0.79, 0.25], I^2^=NA | One study, N=15, SMD=-0.04, 95%CI[-1.05, 0.98], I^2^=NA | One study, N=15, SMD=0.18, 95%CI[-0.84, 1.20], I^2^=NA |
| **Anticonvulsants** | | | | |
| Topiramate | | One study, N=30, SMD=-0.32, 95%CI[-1.04, 0.40], I^2^=NA | One study, N=30, SMD=-0.49, 95%CI[-1.22, 0.24], I^2^=NA | One study, N=30, SMD=-0.36, 95%CI[-1.08, 0.37], I^2^=NA |
| Divalproex | | Two studies, N=110, SMD=0.35, 95%CI[-0.80, 1.50], I^2^=85% | Two studies, N=110, SMD=0.03, 95%CI[-0.34, 0.41], I^2^=0% | Two studies, N=110, SMD=0.23, 95%CI[-0.14, 0.61], I^2^=0% |
| **MAOI** | | | | |
| Phenelzine | | One study, N=37, SMD=-0.81, 95%CI[-1.49, -0.14], I^2^=NA | NR | One study, N=37, SMD=-1.07, 95%CI[-1.77, -0.38], I^2^=NA |
| **N-acetylcysteine** | | | | |
| N-acetylcysteine | | One study, N=27, SMD=-0.61, 95%CI[-1.38, 0.17], I^2^=NA | One study, N=27, SMD=-0.15, 95%CI[-0.91, 0.60], I^2^=NA | One study, N=27, SMD=-0.20, 95%CI[-0.96, 0.55], I^2^=NA |
| **Reversible cholinesterase inhibitor** | | | | |
| Rivastigmine | | One study, N=24, SMD=-0.41, 95%CI[-1.22, 0.40], I^2^=NA | One study, N=24, SMD=-0.33, 95%CI[-1.13, 0.48], I^2^=NA | One study, N=24, SMD=-0.18, 95%CI[-0.98, 0.62], I^2^=NA |
| **SARIs** | | | | |
| Nefazodone | | One study, N=41, SMD=-0.13, 95%CI[-0.77, 0.51], I^2^=NA | One study, N=41, SMD=-0.34, 95%CI[-0.98, 0.30], I^2^=NA | One study, N=41, SMD=-0.15, 95%CI[-0.79, 0.48], I^2^=NA |
| **SSRIs** | | | | |
| Citalopram | | One study, N=40, SMD=-0.92, 95%CI[-1.57, -0.26], I^2^=NA | One study, N=40, SMD=-1.06, 95%CI[-1.73, -0.39], I^2^=NA | One study, N=40, SMD=0.13, 95%CI[-0.49, 0.75], I^2^=NA |
| Sertraline | | Two studies, N=112, SMD=-0.59, 95%CI[-0.97, -0.21], I^2^=0% | Two studies, N=112, SMD=-0.61, 95%CI[-0.99, -0.23], I^2^=0% | Two studies, N=112, SMD=-0.79, 95%CI[-1.17, -0.40], I^2^=30% |
| **TCAs** | | | | |
| Amitriptyline | | One study, N=33, SMD=-0.90, 95%CI[-1.62, -0.18], I^2^=NA | NR | One study, N=33, SMD=-0.75, 95%CI[-1.46, -0.04], I^2^=NA |
| Imipramine | | One study, N=41, SMD=-0.25, 95%CI[-0.87, 0.37], I^2^=NA | NR | One study, N=41, SMD=-0.19, 95%CI[-0.80, 0.43], I^2^=NA |
| **α2A receptor agonist** | | | | |
| Guanfacine | | One study, N=35, SMD=-0.38, 95%CI[-1.05, 0.29], I^2^=NA | One study, N=35, SMD=-0.23, 95%CI[-0.89, 0.44], I^2^=NA | One study, N=35, SMD=-0.10, 95%CI[-0.77, 0.56], I^2^=NA |
| **Active drug VS. Active drug** | | | | |
| Phenelzine | Imipmamine | One study, N=42, SMD=-0.56, 95%CI[-1.18, 0.06], I^2^=NA | NR | One study, N=42, SMD=-0.64, 95%CI[-1.27, -0.02], I^2^=NA |

**Note:** AAS: Atypical antipsychotics, GABA agonists: G-aminobutyric acid agonists, MAOI: Monoamine oxidase inhibitor, SARIs: Serotonin antagonist and reuptake inhibitors, SMSs: Serotonin modulator and stimulators, SSRIs: Selective serotonin reuptake inhibitors, TCAs: Tricyclic antidepressants, SMD: Standardized mean difference, RR: Relative risk, CI: Confidence interval, NA: Not applicable, NR: Not reported.

**Supplementary Table 2** Subgroup analysis of comorbidities in all outcomes

| **All outcomes** | **ALL** | | | |  | **Have comorbidity** | | | |  | **No comorbidity** | | | |
| --- | --- | --- | --- | --- | --- | --- | --- | --- | --- | --- | --- | --- | --- | --- |
|  | **SMD/RR** | **95%CI** | **P for SMD/RR** | **I^2^** |  | **SMD/RR** | **95%CI** | **P for SMD/RR** | **I^2^** |  | **SMD/RR** | **95%CI** | **P for SMD/RR** | **I^2^** |
| **Primary outcomes** | | | | | | | | | | | | | | |
| CAPS | -0.23 | [-0.40, -0.06] | <0.05 | 73% |  | -0.16 | [-0.29, -0.04] | <0.05 | 29% |  | -0.22 | [-0.46, 0.01] | 0.06 | 78% |
| Acceptability | 1.03 | [0.89, 1.21] | 0.10 | 0% |  | 1.23 | [0.94, 1.59] | 0.90 | 0% |  | 0.93 | [0.77, 1.13] | 0.45 | 1% |
| Response | 1.34 | [0.97, 1.84] | 0.48 | 55% |  | 2.30 | [0.52, 10.15] | 0.27 | 79% |  | 1.29 | [0.88, 1.88] | 0.16 | 50% |
| Frequencies of complications | 1.62 | [1.20, 2.20] | <0.05 | 0% |  | 1.55 | [0.99, 2.42] | 0.05 | 0% |  | 1.68 | [1.11, 2.56] | <0.05 | 0% |
| **Secondary outcomes** | | | | | | | | | | | | | | |
| Avoidance | -0.26 | [-0.45, -0.07] | <0.05 | 52% |  | 0.00 | [-0.19, 0.19] | 0.92 | 7% |  | -0.31 | [0.54, -0.08] | <0.05 | 53% |
| Hyper-arousal | -0.31 | [-0.46, -0.16] | <0.05 | 35% |  | -0.35 | [-0.74, 0.04] | 0.86 | 43% |  | -0.31 | [-0.49, -0.14] | <0.05 | 39% |
| Re-experiencing | -0.31 | [-0.43, -0.19] | <0.05 | 35% |  | -0.25 | [-0.43, -0.07] | <0.05 | 27% |  | -0.36 | [-0.52, -0.20] | <0.05 | 39% |

**Note:** CAPS: Clinician-Administered PTSD Scale, SMD: Standardized mean difference, RR: Relative risk, CI: Confidence interval.

**Supplementary Table 3** Subgroup analysis of previous treatment in all outcomes

| **All outcomes** | **ALL** | | | | |  | | **Previous medication** | | | | |  | | | **Single drug** | | | | |
| --- | --- | --- | --- | --- | --- | --- | --- | --- | --- | --- | --- | --- | --- | --- | --- | --- | --- | --- | --- | --- |
|  | **SMD/RR** | | **95%CI** | **P for SMD/RR** | **I^2^** |  | **SMD/RR** | | **95%CI** | **P for SMD/RR** | **I^2^** | |  | | **SMD/RR** | | | **95%CI** | **P for SMD/RR** | **I^2^** |
| **Primary Outcomes** | | | | | | | | | | | | | | | | | | | | |
| CAPS | -0.23 | | [-0.40, -0.06] | <0.05 | 73% |  | -0.08 | | [-0.37, 0.21] | 0.57 | 74% |  | | -0.32 | | | [-0.53, -0.12] | | <0.05 | 72% |
| Acceptability | 1.03 | | [0.89, 1.21] | 0.68 | 0% |  | 0.98 | | [0.72, 1.33] | 0.89 | 0% |  | | 1.05 | | | [0.88, 1.26] | | 0.57 | 16% |
| Response | 1.34 | | [0.97, 1.84] | 0.74 | 55% |  | 4.86 | | [1.58, 14.99] | <0.05 | 0% |  | | 1.19 | | | [0.90, 1.57] | | 0.23 | 44% |
| Frequencies of complications | 1.67 | | [1.21, 2.31] | <0.05 | 0% |  | 2.59 | | [1.26, 5.33] | <0.05 | 0% |  | | 1.48 | | | [1.03, 2.13] | | <0.05 | 0% |
| **Secondary outcomes** | | | | | | | | | | | | | | | | | | | | |
| Avoidance | | -0.26 | [-0.45, -0.07] | <0.05 | 52% |  | -0.04 | | [-0.47, 0.38] | 0.84 | 68% |  | | | -0.34 | | | [-0.54, -0.13] | <0.05 | 45% |
| Hyper-arousal | | -0.31 | [-0.46, -0.16] | <0.05 | 35% |  | -0.02 | | [-0.28, 0.24] | 0.87 | 0% |  | | | -0.46 | | | [-0.64, -0.27] | <0.05 | 23% |
| Re-experiencing | | -0.31 | [-0.43, -0.19] | <0.05 | 35% |  | -0.02 | | [-0.28, 0.24] | 0.87 | 0% |  | | | -0.41 | | | [-0.53, -0.25] | <0.05 | 34% |

**Note:** CAPS: Clinician-Administered PTSD Scale, SMD: Standardized mean difference, RR: Relative risk, CI: Confidence interval.

**Supplementary Table 5** Subgroup analysis of different battlefields in all outcomes

| **All outcomes** | **ALL** | | | |  | **OEF＆OIF** | | | |  | **Vietnam War** | | | |
| --- | --- | --- | --- | --- | --- | --- | --- | --- | --- | --- | --- | --- | --- | --- |
|  | **SMD/RR** | **95%CI** | **P for SMD/RR** | **I^2^** |  | **SMD/RR** | **95%CI** | **P for SMD/RR** | **I^2^** |  | **SMD/RR** | **95%CI** | **P for SMD/RR** | **I^2^** |
| **Primary outcomes** | | | | | | | | | | | | | | |
| CAPS | -0.24 | [-0.60, 0.12] | 0.19 | 85% |  | -0.5 | [-1.09, 0.10] | 0.10 | 91% |  | -0.03 | [-0.47, 0.41] | 0.89 | 74% |
| Acceptability | 0.92 | [0.71, 1.21] | 0.57 | 0% |  | 0.75 | [0.33, 1.72] | 0.50 | 0% |  | 0.96 | [0.72, 1.27] | 0.76 | 37% |
| Response | 1.35 | [0.87. 2.09] | 0.18 | 67% |  | NA | NA | NA | NA |  | 1.35 | [0.87. 2.09] | 0.18 | 67% |
| Frequencies of  complications | 2.35 | [1.08, 5.10] | <0.05 | 0% |  | 2.47 | [0.49, 12.36] | 0.27 | 0% |  | 2.3 | [0.95, 5.58] | 0.06 | 0% |
| **Secondary outcomes** | | | | | | | | | | | | | | |
| Avoidance | -0.46 | [-0.69, -0.24] | <0.05 | 36% |  | -0.76 | [-1.15, -0.37] | <0.05 | 0% |  | -0.31 | [-0.59, -0.04] | <0.05 | 30% |
| Hyper-arousal | -0.6 | [-1.11, -0.08] | <0.05 | 67% |  | -0.83 | [-1.22, -0.44] | <0.05 | 0% |  | -0.31 | [-1.36, 0.74] | 0.56 | 82% |
| Re-experiencing | -0.51 | [-0.75, -0.27] | <0.05 | 0% |  | -0.56 | [-1.02, -0.10] | <0.05 | 0% |  | -0.48 | [-0.77, -0.20] | <0.05 | 18% |

**Note:** CAPS: Clinician-Administered PTSD Scale, SMD: Standardized mean difference, RR: Relative risk, CI: Confidence interval, OEF＆OIF: Operation Enduring Freedom and Operation Iraqi Freedom, NA: Not applicable.

**Supplementary Table 6** Subgroup analysis of different acting receptors in primary outcomes

| **Acting receptor** | **Change in Total PTSD Symptoms Scale** | | | |  | **Acceptability** | | | |  | **Response** | | | |  | **Frequencies of complications** | | | |
| --- | --- | --- | --- | --- | --- | --- | --- | --- | --- | --- | --- | --- | --- | --- | --- | --- | --- | --- | --- |
|  | **SMD** | **95%CI** | **P for SMD** | **I^2^** |  | **RR** | **95%CI** | **P for RR** | **I^2^** |  | **RR** | **95%CI** | **P for RR** | **I^2^** |  | **RR** | **95%CI** | **P for RR** | **I^2^** |
| **5-HT receptor** | -0.23 | [-0.67, 0.22] | 0.13 | 84% |  | 0.89 | [0.69, 1.15] | 0.24 | 23% |  | 1.29 | [0.88, 1.88] | 0.15 | 50% |  | 1.45 | [0.88, 2.4] | 0.26 | 0% |
| **Cholinesterase receptor** | -0.36 | [-1.17, 0.45] | 0.26 | NA |  | 1.00 | [0.02, 46.7] | 0.82 | NA |  | NA | NA | NA | NA |  | 1.00 | [0.02, 46.7] | 0.71 | NA |
| **Connexin 43** | -0.54 | [-1.31, 0.23] | 0.19 | NA |  | 1.57 | [0.44, 5.6] | 0.32 | NA |  | NA | NA | NA | NA |  | 0.95 | [0.02, 45.26] | 0.67 | NA |
| **Dopamine receptor** | -0.25 | [-0.61, 0.11] | 0.22 | 48% |  | 0.94 | [0.67, 1.31] | 0.23 | 10% |  | 2.7 | [0.34, 21.53] | 0.65 | NA |  | 1.95 | [0.95, 3.99] | 0.42 | 0% |
| **GABA receptors** | -0.53 | [-1.09, 0.02] | 0.26 | 80% |  | 1.26 | [0.81, 1.94] | 0.33 | 0% |  | NA | NA | NA | NA |  | 2.64 | [1.05, 6.65] | <0.05 | 0% |
| **Glucocorticoid receptor** | 0.09 | [-0.45, 0.62] | 0.31 | NA |  | 1.97 | [0.85, 4.57] | 0.41 | NA |  | NA | NA | NA | NA |  | NA | NA | NA | NA |
| **NMDA receptors** | 0.10 | [-0.12, 0.31] | 0.14 | 31% |  | 1 | [0.14, 6.99] | 0.51 | 0% |  | NA | NA | NA | NA |  | NA | NA | NA | NA |
| **α1 receptor** | 0.02 | [-0.17, 0.2] | 0.12 | 0% |  | 0.88 | [0.58, 1.34] | 0.18 | 0% |  | 6 | [1.54, 23.44] | 0.59 | NA |  | 1.22 | [0.61, 2.44] | 0.34 | 0% |
| **α2 receptor** | -0.34 | [-0.62, -0.06] | <0.05 | 31% |  | 1.57 | [0.78, 3.18] | 0.38 | 0% |  | 1.04 | [0.82, 1.32] | 0.18 | NA |  | 8.17 | [0.44, 151.84] | 0.93 | NA |

**Note:** NMDA: N−methyl−d−aspartate, GABA: Gamma aminobutyric acid, 5-HT: 5-hydroxytryptamine, RR: Relative risk, SMD: Standardized mean difference, CI: Confidence interval, NA: Not applicable.

**Supplementary Table 7** Subgroup analysis of different acting receptors in secondary outcomes

| **Acting receptor** | **Avoidance** | | | |  | **Hyper-arousal** | | | |  | **Re-experiencing** | | | |
| --- | --- | --- | --- | --- | --- | --- | --- | --- | --- | --- | --- | --- | --- | --- |
|  | **SMD** | **95%CI** | **P for SMD** | **I^2^** |  | **SMD** | **95%CI** | **P for SMD** | **I^2^** |  | **SMD** | **95%CI** | **P for SMD** | **I^2^** |
| **5-HT receptor** | -0.53 | [-0.77，-0.30] | <0.05 | 0% |  | -0.54 | [-0.86, -0.21] | <0.05 | 0% |  | -0.62 | [-0.86, -0.39] | <0.05 | 24% |
| **Cholinesterase receptor** | -0.41 | [-1.22, 0.40] | 0.29 | NA |  | -0.33 | [-1.13, 0.48] | 0.37 | NA |  | -0.18 | [-0.98, 0.62] | 0.51 | NA |
| **Connexin 43** | -0.61 | [-1.38, 0.17] | 0.31 | NA |  | -0.15 | [-0.91, 0.60] | 0.33 | NA |  | -0.2 | [-0.96, 0.55] | 0.49 | NA |
| **Dopamine receptor** | 0.09 | [-0.31, 0.48] | 0.24 | 65% |  | -0.47 | [-0.95, 0.01] | 0.11 | 50% |  | -0.35 | [-0.55, -0.16] | <0.05 | 30% |
| **GABA receptors** | -0.28 | [-0.72, 0.16] | 0.29 | 53% |  | -0.3 | [-0.84, 0.24] | 0.23 | 68% |  | 0.00 | [-0.32, 0.31] | 0.33 | 0% |
| **Glucocorticoid receptor** | NA | NA | NA | NA |  | NA | NA | NA | NA |  | NA | NA | NA | NA |
| **NMDA receptors** | NA | NA | NA | NA |  | NA | NA | NA | NA |  | NA | NA | NA | NA |
| **α1 receptor** | 0.11 | [-0.27, 0.48] | 0.11 | 0% |  | 0.04 | [-0.33, 0.42] | 0.26 | 0% |  | 0.05 | [-0.30, 0.41] | 0.24 | 0% |
| **α2 receptor** | -0.38 | [-1.05, 0.29] | 0.42 | 52% |  | -0.23 | [-0.89, 0.44] | 0.29 | NA |  | -0.10 | [-0.77, 0.56] | 0.31 | NA |

**Note:** NMDA: N−methyl−d−aspartate, GABA: Gamma aminobutyric acid, 5-HT: 5-hydroxytryptamine, SMD: Standardized mean difference, CI: Confidence interval, NA: Not applicable

**Supplementary Figure 1** Risk of bias summary

**
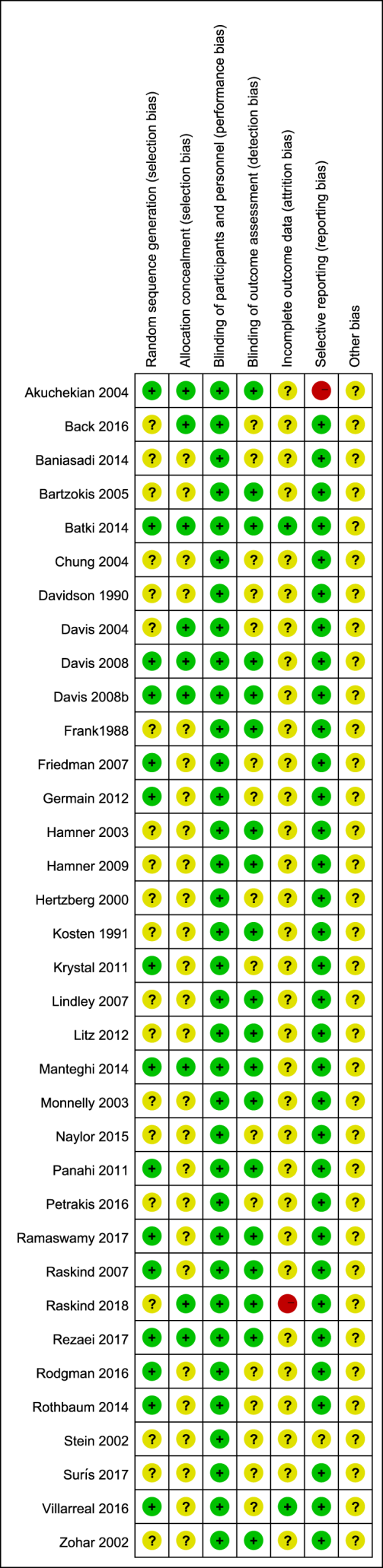
**

**Supplementary Figure 2** Funnel of all outcomes

**
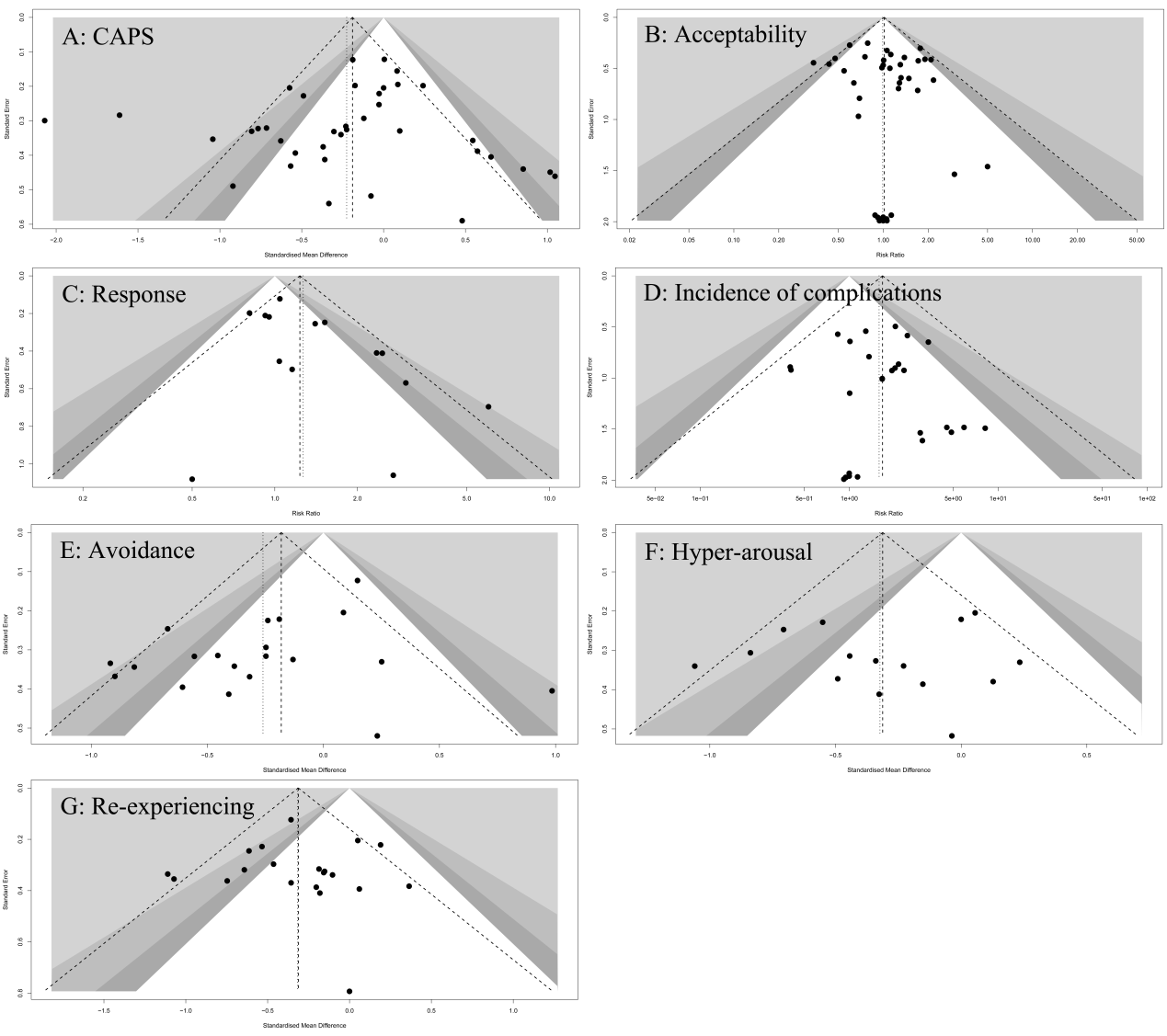
**
